# A reformulation of Murashige and Skoog medium (WPBS medium) improves embryogenesis, morphogenesis and transformation efficiency in temperate and tropical grasses and cereals

**DOI:** 10.1007/s11240-020-01784-8

**Published:** 2020-02-19

**Authors:** S. J. Dalton

**Affiliations:** IBERS, Aberystwyth University, Aberystwyth, Wales, UK

**Keywords:** Embryogenic callus, Transformation, Forage grasses, Model grasses, Energy grasses, Cereals

## Abstract

Slow callus growth is a barrier to efficient genetic transformation in some gramineous species. A reformulation of Murashige and Skoog (MS) medium, with additional magnesium sulphate, potassium phosphate, copper sulphate, proline and glutamine, termed WPBS medium, has been developed which improves all aspects of in vitro culture when compared with MS based media. Embryogenic callus could be produced more rapidly from responsive genotypes of sixteen cereal, forage, model and energy grass species, whether using embryos, shoot tips or proliferated meristems as explants. Three species were not transformed due to contamination or unsuitable explant, but thirteen species were transformed using an identical *Agrobacterium*-mediated transformation, selection and regeneration protocol, including *Avena sativa* and *Oryza sativa*. Readily transformable species such as *Lolium perenne*, *Brachypodium distachyon* and *Festuca arundinacea* and recalcitrant species such as *Lolium temulentum* and *Miscanthus sinensis* were reliably transformed, while two new species *Phalaris arundinacea* and viviparous *Deschampsia cespitosa* were transformed at the first attempt. It is hoped that the use of WPBS media and this general transformation protocol may help to improve the efficiency of grass and cereal transformation.

**Electronic supplementary material:**

The online version of this article (10.1007/s11240-020-01784-8) contains supplementary material, which is available to authorized users.

## Key Message

WPBS medium and a general transformation protocol improves plant growth, embryogenic callus growth, genetic transformation and plant regeneration in a wide range of grass and cereal species.

**Electronic supplementary material:**

The online version of this article (10.1007/s11240-020-01784-8) contains supplementary material, which is available to authorized users.

## Introduction

The induction of fast-growing, embryogenic callus is a pre-requisite for efficient genetic transformation in grasses and cereals and cultures have generally been grown on Murashige and Skoog (1962) medium (Wang et al. 2001). However, several important species are slow growing on this medium and *Miscanthus* species for instance are currently only transformed with low efficiency (Wang et al. 2011; Hwang et al. 2014; Yoo et al. 2018). A model grass transformation system such as that devised for *B. distachyon* (Thole and Vain 2012) produces transformable callus in 6 weeks while most *Miscanthus* explants have barely started to grow by then.

Several compounds have been shown to improve embryogenic callus growth in particular grasses and cereals: magnesium chloride (Holme et al. 1997), silver nitrate (Frame et al. 2002), copper sulphate (Ha et al. 2001; Choi et al. 2001; Thole and Vain 2012), proline (Holme et al. 1997; Frame et al. 2002; Dalton 2013; Patel et al. 2013; Hwang et al. 2014) and glutamine (Zhang et al. 2013; Pawar et al. 2015) or shown to be rapidly depleted in cultures: potassium phosphate (Pepó and Tóth 2003).

These compounds have been assessed and combined in *Miscanthus* media to improve transformation efficiency (Dalton unpub) and positive results using these media with other grass and cereal species has led to the development and refinement of a modified basal medium for grasses and cereals generally (Dalton unpub). This modified MS medium, which contains additional magnesium sulphate, potassium phosphate, copper sulphate, proline and glutamine has been named WPBS medium.

During the development of WPBS medium, a single *Agrobacterium*-mediated transformation protocol was used where possible for all species. However, the explant used for callus induction depends on species. Immature embryos have normally been cultured for callus induction in annual species such as *Brachypodium distachyon* (Thole and Vain 2012), *Lolium temulentum* (Dalton et al. 1999) and *Zea mays* (Frame et al. 2002), while mature embryos were used in *Oryza sativa* (Shri et al. 2013). With *Avena sativa* however, panicles contain few immature embryos at the same suitable stage, but Maqbool et al. (2002) have devised a medium to induce multiple meristems from *A. sativa* shoot tips for direct transformation. Shoot tips containing the apical meristem have been routinely used to induce embryogenic callus from transformable genotypes of perennial, outbreeding grass species for many years (Dalton et al. 1998, 1999, 2003).

For *Agrobacterium*-mediated transformation, the infection and co-cultivation media devised for maize by Frame et al. (2002) were used, but modified by the addition of maltose (Patel et al. 2013) and the omission of myo-inositol, which was shown to reduce *Agrobacterium tumefaciens* infectivity (Zhang et al. 2013). Additional copper sulphate was also omitted as it had been shown to reduce *Agrobacterium* growth (Nawapan et al. 2009). These infection and co-cultivation media have long been used successfully in all routinely transformed species in this laboratory (Dalton unpub.) as has a regeneration medium (RM) devised for *Dicanthium annulatum* (Dalton et al. 2003).

This study used sixteen grass and cereal species to compare WPBS-based media with MS-based media at every stage of this general *Agrobacterium*-mediated transformation protocol.

## Materials and methods

### Preliminary development of WPBS medium

Over one hundred different formulations of MS medium were evaluated quantitively by determining embryogenic callus growth and subsequent shoot regeneration (data not shown). A rationale for developing the medium and a list of the concentrations of each compound tested is shown in Suppl. Text 1 and Suppl. Table 1. The final basal medium was defined and named WPBS medium to celebrate the 2019 centenary of the Welsh Plant Breeding Station. WPBS medium was compared directly against the control MS basal medium at every stage of transformation and was the main objective of this study.

**Table 1:** WPBS medium compared with full strength and ¾ strength macro-elements Murashige and Skoog medium

Ingredient mgl^−1^ and *final molarity mM*	Control medium Modified MS (Duchefa M0245)	MS ¾ salts (Duchefa M0234)	WPBS medium (M0234 with WPBS solution)
CaCl_2_ MW 111	332.02 *3*	249.02 *2.24*	249.02 *2.24*
KH_2_PO_4_ MW 136	170 *1.25*	127.5 *0.94*	277.5 *2.04*
KNO_3_ MW 101	1900 *18.79*	1425 *14.09*	1425 *14.09*
MgSO_4_ MW 120	180.54 *1.5*	136.01 *1.13*	426 *3.54*
NH_4_NO_3_ MW 80	1650 *20.61*	1237.5 *15.46*	1237.5 *15.46*
CuSO_4_ 5H_2_O MW 250	0.025 *0.1 µM*	0.025 *0.1 µM*	0.625 *2.5µM*^a^
Total weight of macro elements	4232	3175	3615
Proline			700
Glutamine			125
Thiamine	1		1
Myo-inositol	100		100^b^
Nicotinic acid	0.5		0.5
Pyridoxine HCl	0.5		0.5
Glycine	2		2
Micro elements	MS	MS	MS^c^
Total molarity (mM)			
Ca	3	2.24	2.24
Cu	0.1	0.1	2.5
K	20.04	15.03	16.13
Mg	1.5	1.13	3.54
NO3	39.4	29.55	29.55
PO4	1.25	0.94	2.04
SO4	1.5	1.13	3.54

^a^Omitted from WPBS-C

^b^Omitted from WPBS-B and WPBS-C by use of Chu’s N6 vitamins

^c^As for Murashige and Skoog except additional CuSO_4_

A single transformation protocol was followed with all sixteen species, using the same *Agrobacterium* infection and co-cultivation media, and plant regeneration and rooting media. The only differences in the experimental protocol between species were the explants used to induce callus, the requirements for growth regulators such as 2,4-D and BAP in callus media and increased proline for *Miscanthus*, and the timing of callus induction regimes.

### Plant material

Selected tissue culture responsive genotypes of perennial out-breeding grasses and responsive lines or cultivars of inbreeding grasses and cereals were used. These were *Agrostis stolinifera* L.[creeping bentgrass] cv Sefton genotype C120SeftonE (Dalton et al. 1998); *Avena sativa* L*.*[oat] cvs Bajka (Gasparis and Nadolska-Orczyk 2015) and Assinaboia; *Brachypodium distachyon* (L.) P.Beauv.[stiff brome] line BD21-3 (Vogel and Hill 2008); viviparous *Deschampsia cespitosa* (L.) P.Beauv.[tufted hairgrass] genotype ABY-Bs3667 (Dalton unpub.); *Festuca arundinacea* Schreb.[tall fescue] cv Aberystwyth S170 genotype CS20BN3 (Dalton et al. 1998; Bettany et al. 2003; Buanafina et al. 2015); *Festuca rubra* L*.*[red fescue] cv Barcrown genotype C123 BarcrownA0 (Dalton unpub.); *Lolium multiflorum* Lam.[Italian ryegrass] cv Trident genotype C38BB13 (Dalton et al. 1998, 1999; Bettany et al. 2003); *Lolium perenne* L.[perennial ryegrass] cv Aberystwyth S23 genotype CS128S23Z (Dalton et al. 1998, 1999); *Lolium temulentum* L.[darnel] genotype ABY-Ba3081 (Dalton and Thomas 1992; Dalton et al. 1999); *Miscanthus sinensis* Andersson genotype Suegen14 (Dalton 2013); *Miscanthus floridulus* genotype ABY-Mb1125 (Dalton 2013); *Miscanthus sacchariflorus* (Maxim.) Franch. genotype Robustus (Dalton 2013); *Oryza sativa* [rice] cvs IR-64 (Shri et al. 2013), IET4786 (Shri et al. 2013) and Italian long grain; *Phalaris arundinacea* L.[reed canary grass] cv Bamse genotypes C416RCG9, C372RCGI9-8, C372RCGI9-10 (Dalton unpub.); *Poa pratensis* L.[smooth-stalked meadow grass] apomictic genotype C8 A24 (Robson et al. 2004); *Zea mays* L.[maize] genotype A188 (Ishida et al. 1996).

### WPBS and MS culture media

The MS control medium used throughout was a modified MS medium (Duchefa M0245) containing 1 mgl^−1^ thiamine. WPBS medium was based on this MS medium, but used three-quarter strength macro-elements and contained extra potassium, phosphate, sulphate, magnesium, copper and thiamine as well as proline and glutamine as a basal medium (Table 1).

For *Agrobacterium*-mediated transformation three WPBS stock solutions were required, one with myo-inositol (WPBS-A) for normal growth, selection and regeneration, one without myo-inositol (WPBS-B) for callus induction and a third (WPBS-C) without myo-inositol and copper sulphate for *Agrobacterium* infection and co-cultivation. For particle bombardment and propagation WPBS-A was used throughout.

The three WPBS compositions A, B and C (Suppl. Table 2) were made as stock solutions for 100 L of final medium (Suppl. Table 3).

**Table 2:** WPBS culture media and explants used in different grass species for normal growth, propagation, callus induction, transformation and regeneration. Control media were made using modified MS medium (Duchefa M0245) instead of WPBS medium. All media were autoclaved for 15 min at 121 °C, solidified with 0.03% Gelrite and adjusted to pH 5.6 unless stated otherwise

Purpose of culture medium and species	Explant	WPBS culture medium per litre
1. Shoot tip culture, rooting and maintenance medium (MSO)	Shoot tips, shoots	WPBS-A, 3% sucrose
2. *Avena* meristem medium (OMM) (*Avena sativa, Lolium perenne,* *L. temulentum, Festuca arundinacea)*	Shoot tips from seedlings or plantlets on MSO	WPBS-A, 3% maltose, 0.5 mg 2,4-D, 2 mg BAP
3. Pre-transformation callus induction media		
* Lolium perenne*	Shoot tips from plantlets on MSO or meristem clusters from OMM	WPBS-B, 3% maltose, 5 mg 2,4-D, 0.5 mg BAP
* Lolium temulentum*	Immature embryos 0.5-1 mm or meristem clusters from OMM	
* Avena sativa*	Meristem clusters from OMM	
* Festuca arundinacea*,	Shoot tips from plantlets on MSO or meristem clusters from OMM	WPBS-B, 3% maltose, 4 mg 2,4-D
* Agrostis stolonifera*, *Deschampsia cespitosa*, *Festuca. rubra*, * Lolium multiflorum*, *Poa pratensis*,	Shoot tips from plantlets on MSO	WPBS-B, 3% maltose, 3 mg 2,4-D
* Phalaris arundinacea*	Shoot tips from plantlets on MSO or meristem clusters from OMM	
* Oryza sativa*	Mature embryos (shoot tips and OMM not successful)	
* Brachypodium distachyon*	Immature embryos 0.2–0.5 mm (shoot tips and OMM not successful)	WPBS-B, 3% maltose, 2.5 mg 2,4-D
* Zea mays*	Immature embryos 1.5-2 mm (shoot tips not successful)	WPBS-B, 3% maltose, 2 mg 2,4-D
4. Transformation media		
* Agrobacterium* infection medium (liquid)^a ^	Immature embryos, calli, cell suspensions	WPBS-C, 6.84% sucrose, 3.6% glucose, 2.5 mg 2,4-D, pH 5.2
* Agrobacterium* co-cultivation medium (liquid)^a^	Immature embryos, calli, cell suspensions	WPBS-C, 6% maltose, 300 mg cysteine, 2.5–5 mg 2,4-D, pH 5.2
Osmotic bombardment medium	Calli, cell suspensions	WPBS-A, 3% maltose, 9% sorbitol, 3 mg 2,4-D
5. Post transformation media		
Callus selection medium	*Agrobacterium* treated or bombarded calli	WPBS-A, 3% sucrose, pH 5.6, growth regulators as for calli above
Regeneration medium (RM)	Calli	WPBS-A, 3% maltose, 1 mg BAP, 1 mg NAA
Rooting medium (MSO)	Shoot tips, shoots	WPBS-A, 3% sucrose
6. *Miscanthus* media where different for *Miscanthus sinensis, M.floridulus, M.sacchariflorus*		
Shoot tip culture, rooting and maintenance medium (liquid) (MS1.5P)	Shoot tips, shoots	WPBS-A, 3% sucrose, 0.8 g proline (1.5 g total)
Propagation medium (liquid)	Shoots	WPBS-A, 3% sucrose, 0.3 mg BAP, 0.8 g proline (1.5 g total)
Pre-transformation callus induction medium	Shoot tips from plantlets on propagation medium	WPBS-B, 3% maltose, 3 mg 2,4-D, 0.1 mg BAP, 0.7 g proline (1.4 g total)
Osmotic bombardment medium	Calli	WPBS-A, 3% maltose, 5.5% mannitol, 5.5% sorbitol, 3 mg 2,4-D
Post-transformation callus selection medium	*Agrobacterium* treated or bombarded calli	WPBS-A, 3% sucrose, 3 mg 2,4-D, 0.1 mg BAP, 0.7 g proline (1.4 g total)

^a^Filter sterilised through 0.2 µM filter

**Table 3:** Comparisons a–f of MS control and WPBS media, callus culture regimes and transformation treatments in *Agrobacterium* mediated transformations of various grass species using genes of interest or *gus A*

Species	Medium and treatment	No. of explants or embryos	Timing	Total weight of callus (g)	GUS or PCR pos plants or (calli)	Plants per g callus	Plants per explant	Significance between plant numbers per replicate dish
(a) Comparison of MS control and WPBS media and different callus culture regimes using *gus A*								
* L. perenne*	MS	9	6, 3, 2, 1 weeks	13.23	30	2.27	3.33	NS
* L. perenne*	WPBS	10	6, 3, 2, 1 weeks	34.01	100	2.94	10	
* L. temulentum*	MS	425	10–12 day	10.81	3 (27)	0.28	0.01	P ≤ 0.01
* L. temulentum*	WPBS	566	10–12 day	17.63	23 (70)	1.3	0.04	
* B. distachyon*	MS	50	3, 1 weeks	4.34	3	0.69	0.06	P ≤ 0.01
* B. distachyon*	WPBS	55	3, 1 weeks	9.25	16	1.73	0.29	
* B. distachyon*	MS	43	3, 2, 1 weeks	27.09	1	0.04	0.02	P ≤ 0.01
* B. distachyon*	WPBS	47	3, 2, 1 weeks	41.09	18	0.44	0.38	
* F. arundinacea*	MS	n/r	6, 3, 2, 1 weeks	3.59	3	0.84	n/r	NS
* F. arundinacea*	WPBS	n/r	6, 3, 2, 1 weeks	6.68	9	1.35	n/r	
* M. sinensis*	MS	9	6, 2, 2, 1 weeks	9.97	0	0	0	NS
* M. sinensis*	WPBS	15	6, 2, 2, 1 weeks	36.73	25	0.68	1.67	
* M. floridulus*	MS	8	6, 2, 2, 1 weeks	12.66	0	0	0	NS
* M. floridulus*	WPBS	25	6, 2, 2, 1 weeks	28.07	1	0.04	0.04	
(b) Comparison of adding or leaving out copper (Cu) in infection and co-cultivation media using *gus A*								
* L. perenne*	MS	4	6, 3, 2, 1 weeks	6.4	14	2.19	3.5	MS and WPBS NS + and − Cu NS
* L. perenne*	MS + Cu	5	6, 3, 2, 1 weeks	6.83	16	2.34	3.2	
* L. perenne*	WPBS-Cu	5	6, 3, 2, 1 weeks	17.51	57	3.26	11.4	
* L. perenne*	WPBS + Cu	5	6, 3, 2, 1 weeks	16.6	43	2.59	8.6	
(c) Comparison of calli derived from shoot tips and OMM meristem tissue using genes of interest								
* F. arundinacea*	WPBS	68	6, 2, 1 weeks	11.22	16	1.43	0.24	P ≤ 0.05
* F. arundinacea*	WPBS OMM	45	8, 1.5 weeks	15.03	13	0.86	0.29	
* L. perenne*	WPBS	74	5, 2, 1 weeks	9.64	17	1.76	0.23	NS
* L. perenne*	WPBS OMM	19	5, 2, 1 weeks	20.05	11	0.55	0.58	
* L. temulentum*	WPBS	1848	4–5, 1 weeks	311.02	4	0.01	0.002	P ≤ 0.01
* L. temulentum*	WPBS OMM	321	3–5, 1 weeks	70.55	12	0.17	0.037	
(d) Comparison of callus culture regimes using genes of interest								
* B. distachyon*	WPBS	124	3, 1 weeks	18.18	29	1.6	0.23	3,1 wk and 3,2,1wk P ≤ 0.05
* B. distachyon*	WPBS	88	4, 1 weeks	27.59	27	0.98	0.31	
* B. distachyon*	WPBS	47	3, 2, 1 weeks	41.09	18	0.44	0.38	
* L. perenne*	WPBS	45	4, 2, 1 weeks	10.51	20	1.9	0.44	NS
* L. perenne*	WPBS	39	5, 2, 1, 1 weeks	40.99	56	1.37	1.44	
(e) Comparison of vacuum treatment (vac) and immersion (imm) of calli using genes of interest								
* L. temulentum*	MS vac	110	10 day	2.63	1 (4)	0.38	0.01	MS and WPBS P ≤ 0.01 Vacuum and immersion NS
* L. temulentum*	MS imm	154	10 day	3.69	1 (5)	0.27	0.01	
* L. temulentum*	WPBS vac	154	10 day	4.71	6 (19)	1.27	0.04	
* L. temulentum*	WPBS imm	220	10 day	6.73	8 (25)	1.19	0.04	
(f) Comparison of heat shock (HS) and vacuum treatment (vac) of calli using genes of interest								
* B. distachyon*	WPBS HS	29	4, 1 weeks	8.25	6	0.73	0.21	P = 0.054
* B. distachyon*	WPBS vac	29	4, 1 weeks	8.25	15	1.82	0.52	

NS not significant, n/r not recorded

It should be noted that WPBS solution is blue when mixed, due to the Biuret reaction between proline and copper sulphate, but this did not affect medium pH or plant growth. Stock solutions were immediately frozen in 40 ml aliquots and were added to media as required before autoclaving. For each litre of final WPBS medium, 20 mls of WPBS-A, B or C stock solution was added to 3.25 g of three-quarter strength macro-elements MS medium without vitamins (Duchefa M0234) and the pH adjusted to 5.6. Media for all purposes and species (Table 2) were then autoclaved at 121 °C for 15 min, except for *Agrobacterium* infection and co-cultivation media, which were filter-sterilised.

### Comparison of WPBS and MS medium for plant growth

*Brachypodium distachyon* and *L. temulentum* seeds and *A. sativa* caryopses from de-husked seed were surface sterilized in 100% commercial sodium hypochlorite bleach (4.47% available chlorine) for 30–60 min, rinsed in sterile water, imbibed overnight at 4 °C and re-sterilised with 20% bleach for 10 min before use. Ten excised mature embryos of *Brachypodium* and *Lolium* and caryopses of *Avena* were cultured per 90 mm Petri-dish (7–12 replicates) on MS and WPBS-A maintenance medium (MSO) (Table 2) and the seedling dry matter determined after 3 weeks (or 6 weeks for *Brachypodium*). Single tillers from in vitro stock plantlets of *M. floridulus* grown in liquid *Miscanthus* maintenance medium (MS1.5P) with MS (3 tillers) and WPBS-A (13 tillers) were weighed after 7 weeks growth. All cultures were grown at 25 °C in continuous light of ~ 100 µE m^−2^ s^−1^.

### Comparison of WPBS and MS media for callus induction

#### Explants used for callus induction

Callus induction from annual grass species and cereals was through immature embryo culture of transformable lines. Callus induction from perennial, outbreeding species was through shoot tip cultures containing the apical meristem from in vitro plantlets of transformable genotypes. Calli could also be induced from proliferating meristematic cultures of some species. Calli were grown at 25 °C in the dark (*Brachypodium* and *Oryza* at 28 °C).

#### Embryo culture of annual grasses and cereals

Spikelets from immature inflorescences of *B. distachyon* and *L. temulentum* stored at 4 °C overnight, were surface sterilized in 100% commercial sodium hypochlorite bleach (4.47% available chlorine) for 20–30 min and rinsed in sterile water. Semi-translucent immature embryos, *Lolium* (0.5–1 mm) and *Brachypodium* (0.2–0.5 mm), were cultured with the scutellum uppermost. Mature embryos of rice were cultured from seed which had been surface-sterilized twice as described.

#### Shoot tip culture of perennial grasses

Shoot tips (0.3–0.5 mm) containing the apical meristem were cultured from sterile in vitro stock plants of perennial grasses, growing on MS or WPBS-A maintenance medium (MSO) or *Miscanthus* propagation medium (Table 2). Calli were grown for about 5 weeks and sub-cultured to fresh medium at least twice before cultures of up to 7 days from the last subculture were used for transformation. Three replicate dishes of twenty *F. arundinacea, L. multiflorum* and *L. perenne* shoot tip calli growing on MS and WPBS-B medium were compared after 31, 51 and 60 days.

#### Proliferating meristem culture of *Avena sativa*

Seedlings were germinated from surface sterilized seed on WPBS-A maintenance medium (MSO). In vitro seedling shoot tips (0.3–0.5 mm) were cultured directly on WPBS-B *Avena* callus induction medium or were first cultured on WPBS-A *Avena* meristem proliferation medium (OMM) (Table 2) to produce additional meristematic tissue for callus induction. Cultures were grown in the light at 25 °C for 5 weeks before sub-culture to fresh medium and were used for up to 2 months. Small meristematic clusters were transferred to callus induction medium (Table 2). This approach was also applicable to *F. arundinacea, L. perenne* and *L. temulentum*. To compare shoot-tips and meristem clusters for *Avena* callus induction, ten shoot tips and ten meristematic clusters were cultured on WPBS-B *Avena* callus medium (5 replicates each). The calli were gathered and weighed at subculture to fresh medium after 33, 46 and 52 days.

### Comparison of WPBS with MS media using different callus induction regimes

Most perennial grasses required a period of at least 4 weeks of initial shoot tip callus induction followed by several subcultures totaling 9 or more weeks (Suppl. Table 4). Immature embryos of the fast-growing annual species *B. distachyon,* were cultured with a 3 week, 2 week, 1 week subculture regime, totaling only 6 weeks in culture before transformation. The rapid growth of *Brachypodium* on WPBS medium led to five simpler regimes being tested to reduce sub-culturing and the total time in culture. At least two experiments were performed with each regime with 5–52 replicate dishes of ~ 25 embryos on MS and WPBS media.

With *L. temulentum*, immature embryo cultures were normally transformed after a few days culture, as in maize (Frame et al. 2002). Replicate dishes of ~ 25 embryos (148 dishes with WPBS medium, 60 dishes with MS medium) were cultured in 21 separate experiments to establish the optimum culture period. The callusing embryos were gathered, weighed and transformed after 6 to 13 days culture.

### Comparison of WPBS with MS media for *Agrobacterium-*mediated transformation and the effect of heat shock, vacuum infiltration and *Agrobacterium* immersion

Calli for *Agrobacterium* transformation were induced on MS or WPBS-B based media containing maltose using various explants (Table 2). Most transformations were with genes of interest (GOI), but some used constructs containing the *gus A* gene. These were *gus A* and *hpt* in pBRACT204, (Harwood et al. 2009), in *Agrobacterium* strain AGL1; *gus A* and *bar* in pTF102 in *Agrobacterium* strain EHA105 (Frame et al. 2002), and *gus A*, *hpt* and *nptII* in pTOK233 in *Agrobacterium* strain LBA4404 (Hiei et al. 1994). For each species, the construct with the most effective selection gene was used.

*Agrobacterium* was grown on solid YEP medium (Frame et al. 2002) for 24 h with antibiotics (25 mgl^−1^ rifampicin and e.g. 50 mgl^−1^ hygromycin) at 25 °C and then suspended at an OD_600_ of 0.6 in MS or WPBS-C based sucrose/glucose infection medium devised for maize (Frame et al. 2002) containing 200 µM acetosyringone and 0.02% Pluronic F68. (Table 2). The culture was shaken gently for 16–20 h at 25 °C to maximise infectivity (Xi et al. 2018).

Callus (1–2 g) or ~ 25 callusing embryos were collected in 2 ml infection medium and heat shocked in a water bath at 43 °C for 3 min before adding 10–15 ml of *Agrobacterium* suspension. For most experiments, the calli were collected in *Agrobacterium* suspension before vacuum infiltration with three short pulses at 711 mm (28 in) Hg. In two experiments with *L. temulentum*, the *Agrobacterium* suspension was simply added to the calli on the callus medium to immerse them for the infection period. Calli were incubated with *Agrobacterium* for 30–40 min and dried on 85 mm filter papers in open Petri-dishes for 20–30 min. The dry calli were co-cultivated for 3 days on three 85 mm filter papers wetted with 3 ml co-cultivation medium containing 200 µM acetosyringone at 25 °C in the dark. Stacks of Petri-dishes were wrapped in clingfilm. The MS or WPBS-C based co-cultivation medium contained 6% maltose as devised for *Lolium* (Patel et al. 2013) with cysteine as used in maize (Frame et al. 2002) (Table 2).

### Comparison of WPBS with MS media for callus selection

After 3 days co-culture with *Agrobacterium*, the calli were transferred to MS or WPBS-A callus selection media (Table 2) with 75% of the final concentration of hygromycin, PPT or paromomycin (Suppl. Table 5). The selection media contained sucrose, as maltose allowed non-transformed calli to survive strong selection in *Brachypodium*. Meropenum (50 mgl^−1^) was used as the most effective antibiotic against *Agrobacterium*, when combined with Timentin (100 mgl^−1^). Calli were cultured in the dark at 25 °C (*Brachypodium* and *Oryza* at 28 °C) for 3–4 weeks.

### Comparison of WPBS with MS media for plant regeneration

Calli from callus selection media were transferred to selective MS or WPBS-A RM regeneration medium (Table 2) with 100% selection concentration (Suppl. Table 5) and grown in the light (~ 100 µE m^−2^ s^−1^) at 25 °C for 3–4 weeks until shoots had regenerated. These were transferred to selective MS or WPBS-A based MSO or liquid MS1.5P *Miscanthus* maintenance/rooting media (Table 2) and grown under the same conditions. Rooted plants were transferred to soil in containment glasshouse or controlled growth room conditions under polythene for 1 week.

### Transformation by particle bombardment

In addition to *Agrobacterium* transformation, calli from different species were also transformed by particle bombardment. Calli were bombarded with 0.6 µm gold particles coated with plasmid DNA, containing various GOI, at 7.5 bar in a Particle Inflow Gun (PIG) (Finer et al. 1992) after 6 h on high osmotic potential medium (9 h on a higher osmotic potential medium for *Miscanthus*) (Table 2), using WPBS-A media only. *A. sativa* calli were co-transformed with GOI and pUBA (Toki et al. 1992). Transgenic *F. arundinacea*, *L. temulentum* and *B. distachyon* calli were re-transformed with GOI and pBKS (Dalton et al. 2011). After 24 h, bombarded calli were transferred to callus selection media with 75% selection concentration (Suppl. Table 5) and thereafter treated as for *Agrobacterium* treated callus, but without Meropenum or Timentin antibiotics.

### Confirmation of gene expression

Most experiments were performed with GOI in collaboration with other researchers and transformed plants were confirmed by PCR, generally of the selective gene. Transformed plants in experiments with the *gus A* constructs were confirmed by survival under 100% selection and GUS expression in leaf samples incubated in X-Gluc solution (Dalton et al. 1998).

### Statistical analysis

T-tests and the Pearson co-efficient correlation between independent means were performed respectively using.

https://www.socscistatistics.com/tests/studentttest/default2.aspx

https://www.socscistatistics.com/tests/pearson/default2.aspx

## Results

### WPBS medium increases plant growth

**Fig. 1:**
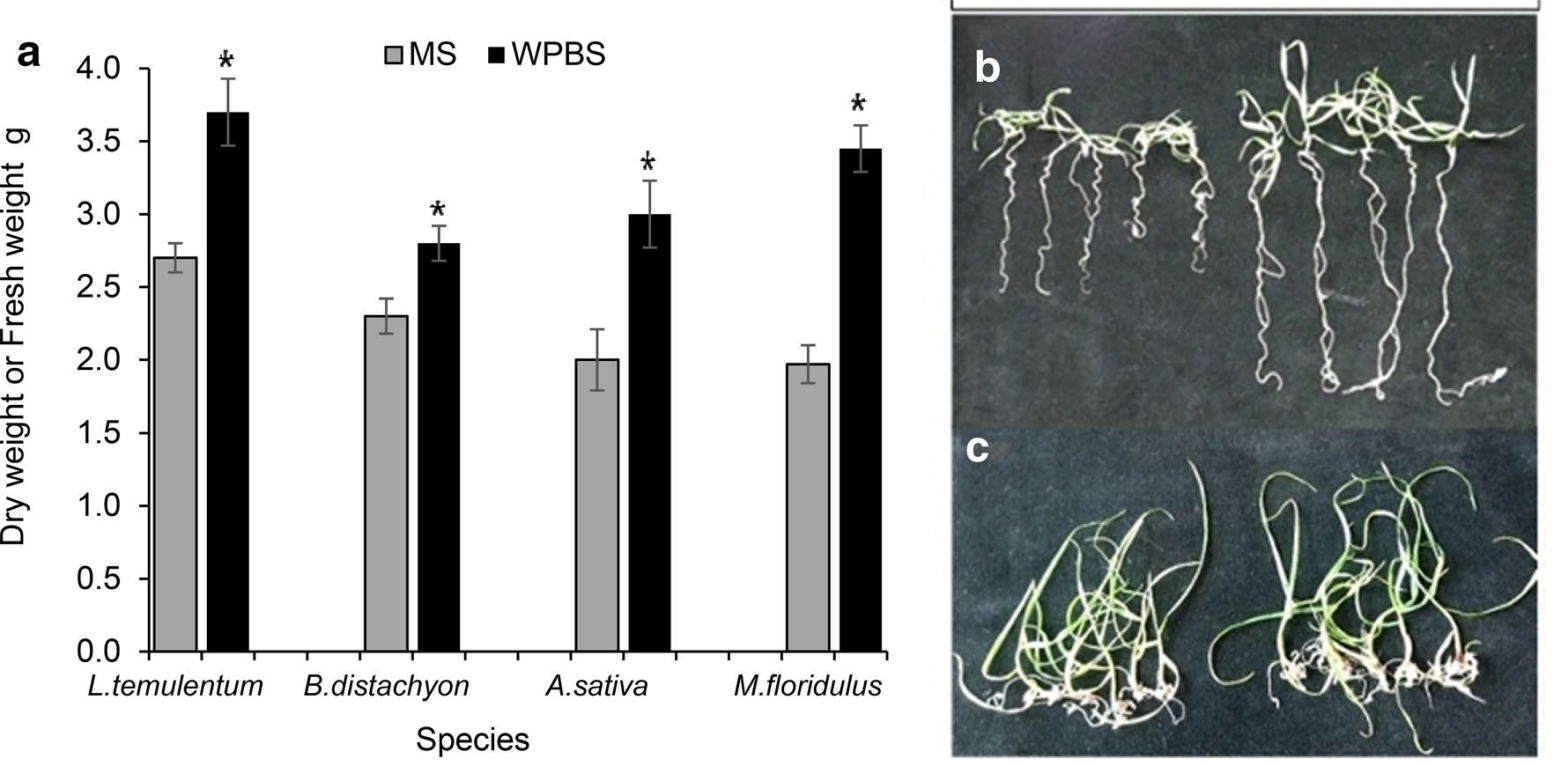
**a **Mean dry weight of 100 seedlings of *Lolium temulentum* and *Avena sativa* after 3 weeks*, Brachypodium distachyon* after 6 weeks and fresh weight of individual plantlets of *Miscanthus floridulus* after 7 weeks cultured on MS or WPBS medium. Mean ± sem. *—means significantly different from MS controls p ≤ 0.01 (*Miscanthus* < 0.025). In vitro seedlings from mature embryos cultured on MS (left) or WPBS ( right) medium **b***Brachypodium distachyon*, **c***Lolium temulentum*

The dry matter of *L. temulentum, B. distachyon* and *A. sativa* seedlings germinated on WPBS medium was significantly increased (P ≤ 0.01) by at least 20% over MS medium (Fig. 1a). Seedlings on WPBS medium had longer roots and were more robust (Fig. 1b, c). Single tillers of *M. floridulus* in WPBS medium also had increased plantlet fresh weight (FW) (P = 0.025), but the mean tiller number per plantlet was not increased.

### WPBS medium increases callus production and reduces culture time

#### *Miscanthus* species

**Fig. 2:**
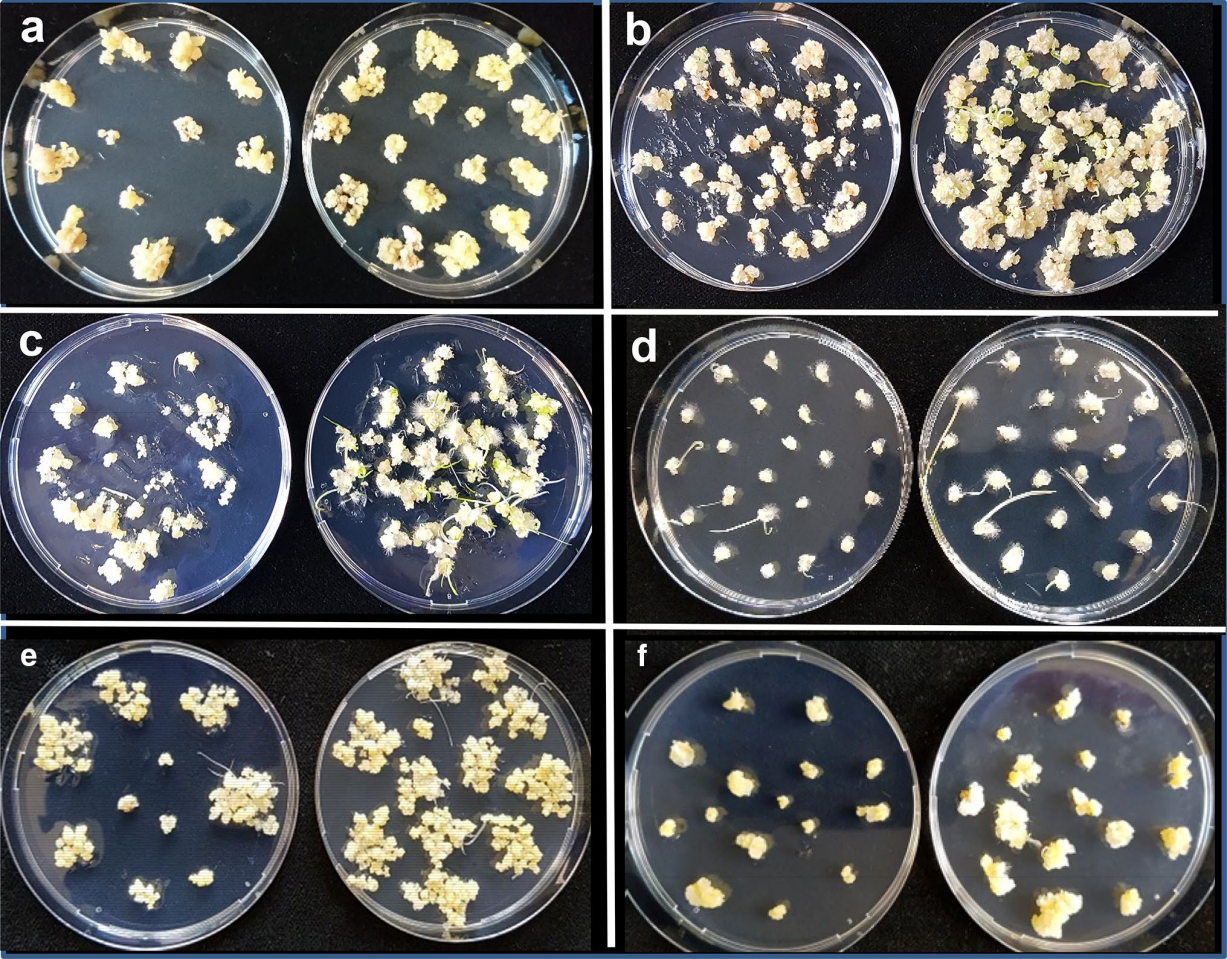
Callus induction on MS control (left) and WPBS media (right): **a***Miscanthus floridulus* shoot tip (ST) calli at 12 weeks,** b***Lolium perenne* ST calli at 12 weeks, **c***Festuca arundinacea* ST calli at 12 weeks, **d***Lolium temulentum* immature embryo (IE) calli at 12 days, **e***Brachypodium distachyon* (IE) calli after 4 weeks, 1 week culture,** f***Avena sativa* oat cv Bajka proliferated meristem calli at 5 weeks

**Fig. 3:**
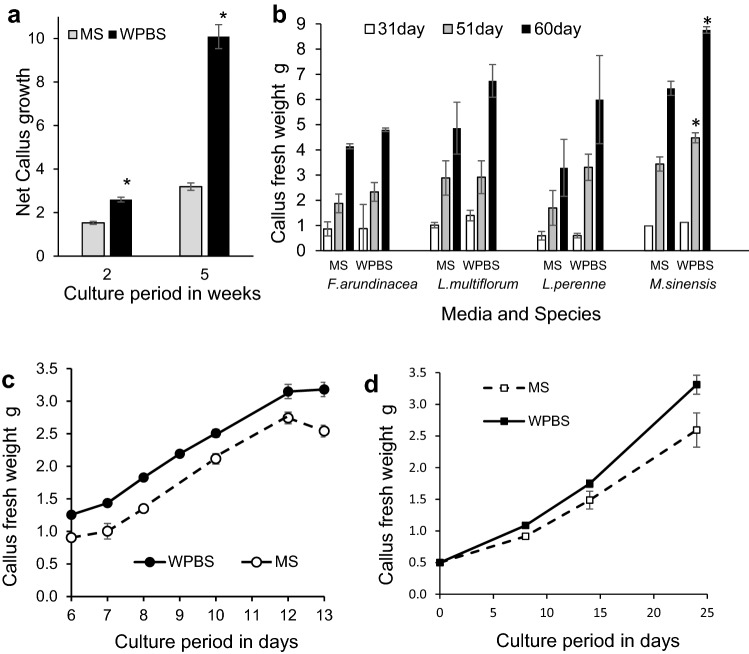
Growth of calli on MS control and WPBS medium **a** Net growth of established *Miscanthus sinensis* calli after two and five weeks culture. Mean ± sem (n = 4 and 15.) *—means significantly different p ≤ 0.01, **b** Growth of calli from 20 shoot tips of *Festuca arundinacea, Lolium multiflorum*, and *L. perenne,* and from 0.18 g *Miscanthus sinensis* callus. Mean ± sem. *—means significantly different from MS controls p ≤ 0.015, **c** Growth of *Lolium temulentum* immature embryos during initial callus initiation. Mean fresh weight of 100 calli ± sem (n = 2–59),** d** Growth of shoot tip-derived *Avena sativa* (oat) callus over 25 days on MS control and WPBS medium. Mean ± sem (n = 3 and 5 respectively)

*Miscanthus* calli of all three species tested were slow to establish embryogenic calli, which were generally produced as a secondary development, following primary watery or friable callus growth (Suppl. Fig. 1a). WPBS medium was originally devised for *Miscanthus* and while there was no difference in the proportion of explants which produced embryogenic callus on MS or WPBS medium (Fig. 2a), the net growth of embryogenic callus was significantly higher (P ≤ 0.01) on WPBS medium with no loss of quality (Fig. 3a). Established callus growth was as high as in other species (Fig. 3b), but a comparison of net growth rates over several experiments showed that sub-culturing more than 0.25 g FW of callus significantly reduced (P ≤ 0.01) callus growth rate (Suppl. Fig. 2a).

#### Other perennial grass species

Callus growth on WPBS medium was also increased in *L. perenne* (Fig. 2b), *F. arundinacea* (Fig. 2c) and *L. multiflorum*, compared with MS medium, but statistically significant differences could not be established due to large variations between replicate dishes (Fig. 3b). Callus induction in perennial species was generally slower than in annual species (Suppl. Fig. 3).

#### *Lolium temulentum*

The FW of calli from immature embryos was significantly higher at every time point over 6–13 days when cultured on WPBS medium than on MS medium (P ≤ 0.01) (Figs. 2d, 3c). Growth rates slowed by day 13, but even after 5 weeks the difference in growth was notable (Suppl. Fig. 1b).

#### *Brachypodium distachyon*

**Fig. 4:**
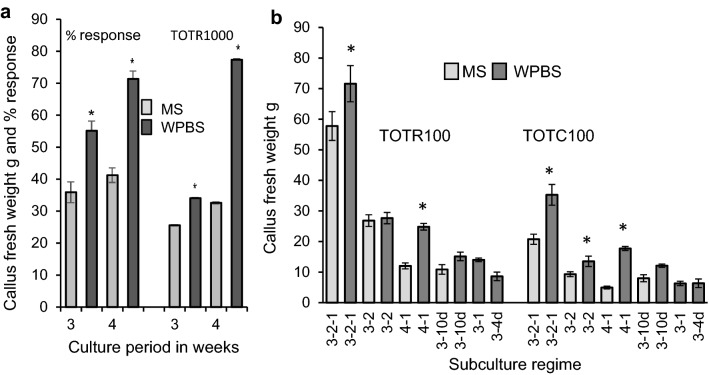
**a **The effect of a three or four week initial culture period on the percentage of *Brachypodium distachyon* immature embryos developing embryogenic callus and the mean fresh weight of callus from 1000 responsive embryos (TOTR1000) on MS and WPBS medium. Means ± sem, * indicates means that are significantly different from MS controls P ≤ 0.01. **b** Growth of embryogenic callus derived from immature embryos *of Brachypodium distachyon* under different subculture regimes on MS or WPBS medium. Mean fresh weight of 100 responsive (TOTR100) or cultured embryos (TOTC100) Means ± sem, *indicates means that are significantly different from MS controls P ≤ 0.05

Only a proportion of cultured immature embryos produced embryogenic callus and at 3 weeks, this was 55% of embryos on WPBS medium, compared with 36% on MS medium (P ≤ 0.01) (Fig. 4a, Suppl. Fig. 1c). By 4 weeks this had increased to 71% of embryos on WPBS medium (P ≤ 0.01), and 41% on MS medium (NS). The FW of embryogenic callus was also significantly higher on WPBS medium after 3 and 4 weeks and increased significantly over the final week (all P ≤ 0.01).

Immature embryos were normally cultured using a three-two-one-week (3-2-1) subculture regime with two subcultures. The final FW of callus from 100 cultured embryos on WPBS medium was nearly 70% higher (P ≤ 0.01) than on MS medium (Fig. 4b). This was partly due to a higher proportion of embryos responding on WPBS medium. However, the FW of callus from 100 responsive embryos was also 24% higher (P ≤ 0.01). At least half of the growth occurred during the last week of culture and net growth was found to be negatively correlated (R =  − 0.7, P ≤ 0.01) with the callus inoculation density. However, despite a higher inoculation density, embryogenic callus cultured on WPBS medium had a higher net growth (2.6-fold) than on MS medium (2.1-fold).

The increase in growth using WPBS medium led to shorter regimes with a single subculture being tested. The amount of callus produced on WPBS medium was significantly higher (P ≤ 0.05) than on MS medium in every regime compared (Fig. 4b). A four-one-week (4-1) regime allowed more embryos to develop and produced 17.8 g of embryogenic callus per 100 cultured embryos (Figs. 2e, 4b). This approached the 20.8 g of callus produced by the control MS three-two-one-week regime, but with only one subculture.

Shoot tips from sterile seedlings were also cultured to induce callus, but only 10% responded and the calli after a five-two-one-week regime were not successfully transformed.

#### *Avena sativa*

Shoot apical meristems from sterile seedlings were easier to culture than immature embryos. Shoot-tip-derived calli on WPBS based medium grew faster than those on MS medium (P = 0.04–0.10) (Fig. 3d).

**Fig. 5:**
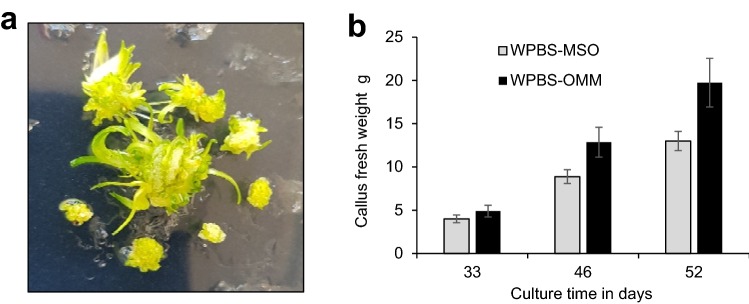
**a **Proliferating meristems of *Avena sativa* (oat) on WPBS-OMM medium,** b** Growth of callus after initiation from shoot tips of seedlings on WPBS-MSO medium or meristem clusters from shoot tips precultured on WPBS-OMM media. Mean fresh weight from 100 shoot tips or meristem clusters, Mean ± sem (n = 5)

WPBS based *Avena* meristem medium (OMM) (Table 2) was used to proliferate the shoot meristems (Fig. 5a) before culture on callus induction medium (Fig. 2f). Meristem-cluster-derived calli grew faster than shoot-tip-derived calli with each subculture (Fig. 5b) (P = 0.28 at 33 days, P = 0.07 at 46 days, P = 0.06 at 52 days). A major benefit of this method was the abundance of meristematic tissue for culture. The OMM medium was also successful with *F. arundinacea, L. perenne* (both P ≤ 0.01), *L. temulentum* and *P. arundinacea* (Suppl. Fig. 1d), but not with *B. distachyon* and *O. sativa* (data not shown).

#### *Oryza sativa* and *Zea mays*

In *O. sativa*, seedling shoot-tip derived calli from all three varieties used were slower growing and less embryogenic than calli from mature embryos, (Suppl. Fig. 1e). Large differences in callus growth were found beween WPBS and MS media from 4 to 10 weeks, especially with mature-embryo-derived calli (Suppl. Fig. 1f), but the number of cultures studied was too small to establish many significant differences (Suppl. Fig. 2b).

In *Z. mays*, immature embryo (Suppl. Fig. 1g) and seedling shoot-tip derived callus of A188 grew well on WPBS medium over 5 weeks. However only immature embryos produced regenerable type II embryogenic callus suitable for transformation.

### WPBS medium increases plant regeneration and rooting

**Fig. 6:**
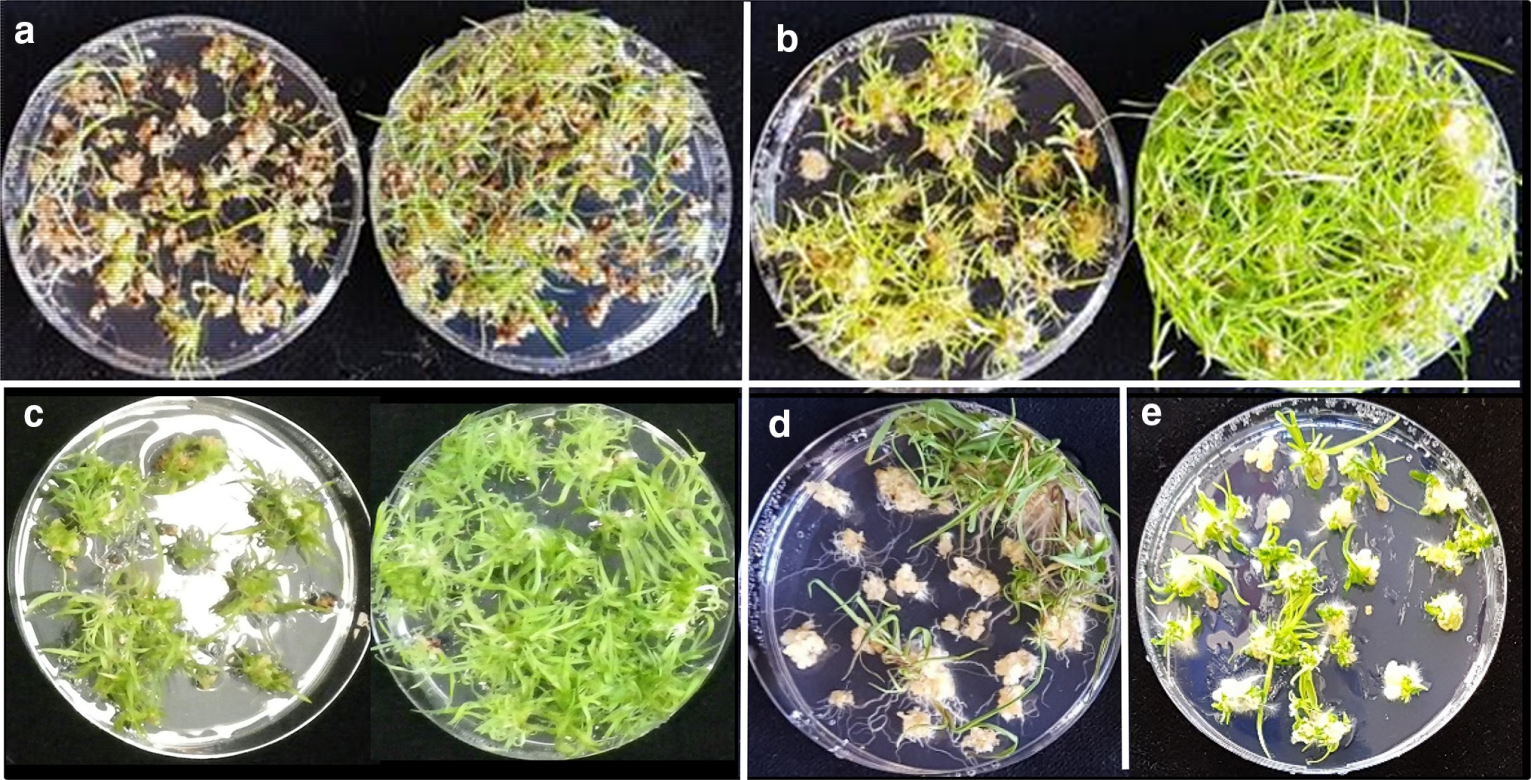
Plant development on MS control (left) and WPBS (right) RM regeneration medium: **a***Brachypodium distachyon*, ***b**** Lolium perenne*, **c***Miscanthus sinensis* and plant regeneration on WPBS RM only: **d ***Phalaris arundinacea*, **e***Avena sativa*

Embryogenic calli from the responsive genotypes of all the species tested, regenerated well on the control MS RM regeneration medium, but in experiments with *B. distachyon, L. perenne* and *M. sinensis,* regeneration was even more frequent on WPBS-A RM medium (Fig. 6a, b, c). *M. sinensis* and *M. floridulus* callus cultures remained regenerative for at least 8 months and albino shoots were very rarely produced. Shoot-tip-derived callus of *P. arundinacea* (Fig. 6d) and proliferated-meristem-derived callus of *A. sativa* (Fig. 6e) regenerated vigorously on the same medium, as did mature-embryo-derived callus of *O. sativa* and immature-embryo-derived callus of *Z. mays*. However, shoot-tip-derived callus of both species was less embryogenic and *O. sativa* callus regenerated poorly (Suppl. Fig. 1h), while *Z. mays* callus turned green but did not regenerate. Anther-derived calli of the three *Miscanthus* species and *A.sativa* also regenerated on WPBS RM medium (Suppl. Fig. 1i, j).

### WPBS medium increases *Agrobacterium* mediated transformation efficiency

In all direct comparisons of transformations of six species between MS and WPBS media, the amount of callus produced and the transformation efficiency, in terms of plants per gram of callus, or plants per explant, was always higher when WPBS media were used (Table 3a, Suppl. Table 6a). Overall, the percentage improvements in transformation efficiency (plants per gram callus) of the six species (Table 3a) comparing WPBS and MS control media was significant (P = 0.02).

#### *Miscanthus* species

**Fig. 7:**
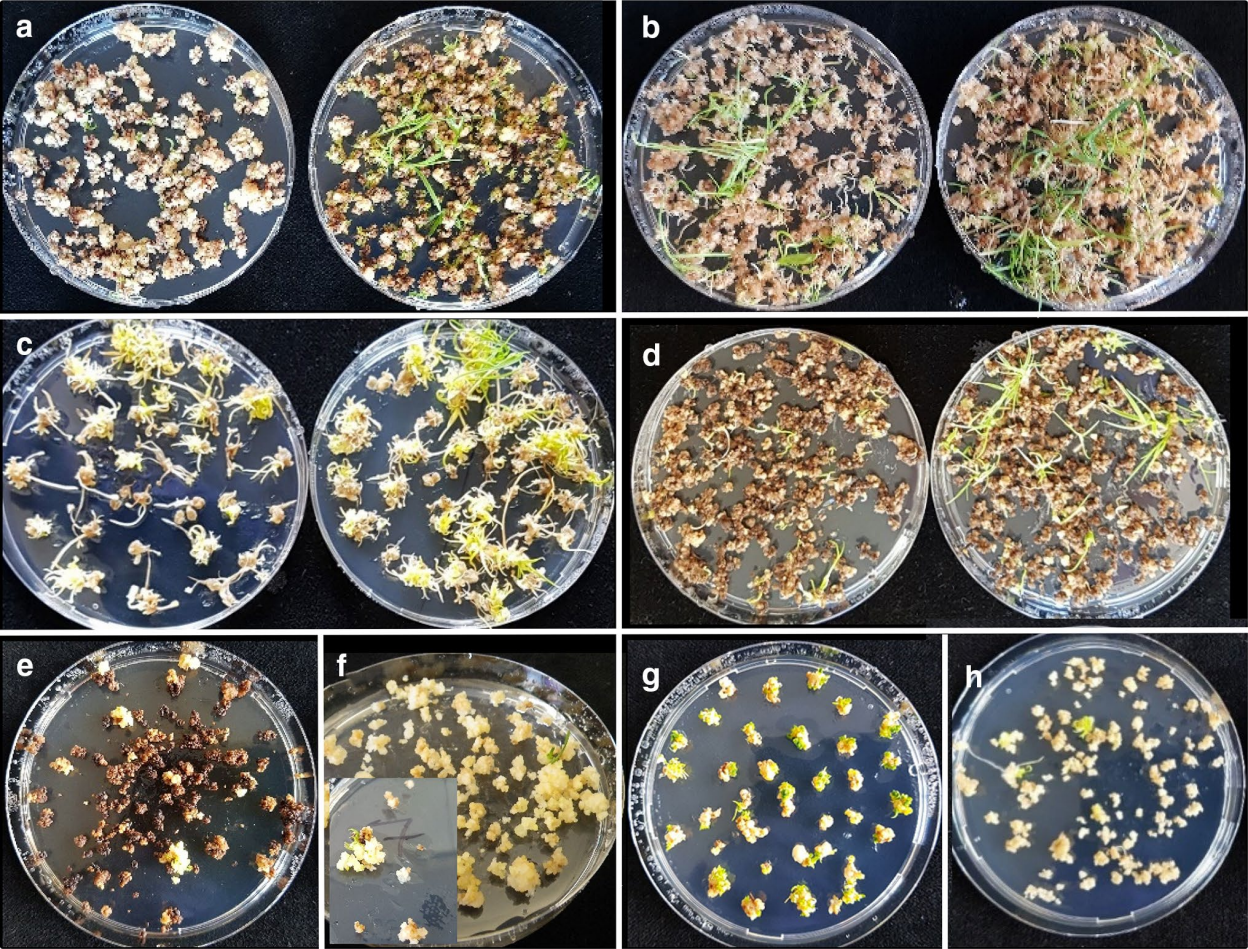
Regeneration of transformed plants under hygromycin selection on MS control (left) and WPBS RM medium (right): **a***Miscanthus sinensis*, **b***Lolium perenne*, **c***Lolium temulentum*, **d***Brachypodium distachyon*, Regeneration on WPBS RM medium only: **e***Phalaris arundincaea* with hygromycin and **f** with paromomycin, **g***Deschampsia cespitosa*, **h***Agrostis stolonifera*

With improved callus growth rate, transformation by *Agrobacterium* as well as bombardment became possible, though at a low frequency except in the most responsive *M. sinensis* genotype, Suegen14 and only by using WPBS media (Table 3a, Suppl. Table 6a, g). Hygromycin selection of calli was clear-cut in *M. sinensis* transformed with the plasmid construct pBRACT204 (Fig. 7a), but in regenerated plants (Suppl. Fig. 6a) GUS activity was restricted to stomata and leaf hairs. GUS activity was stronger and more wide-spread in mature leaves of *M. floridulus* (Suppl. Figs. 5a, 6b), but hygromycin resistant plantlets of *M. sacchariflorus* (Suppl. Fig. 6b) did not express GUS in any cells.

#### *Lolium perenne*

Significantly more GUS expressing plants were recovered from callus from the same number of explants using WPBS compared with MS media (Table 3a, Fig. 7b, Suppl. Fig. 5b). In this experiment, additional copper sulphate in infection and co-cultivation media appeared to reduce efficiency in WPBS grown cultures (Table 3b, Suppl. Fig. 6c) and although the number of plants transformed per replicate (1–2 g callus) was not statistically significant, it was not beneficial and was removed. Although not directly compared, using the *Avena* meristem medium (OMM) to proliferate meristematic tissue in *L. perenne* (and *F. arundinacea*) also improved transformation efficiency in terms of transformants per explant, but not transformants per gram of callus (Table 3c). The number of plants produced per replicate *Agrobacterium* treatment was significantly higher in *Festuca* (P ≤ 0.05). In other experiments with *L. perenne*, older calli were shown to have a lower transformation efficiency per g than calli produced in a 4-2-1-week subculture regime (Table 3d).

#### *Lolium temulentum*

Transformation efficiency was low and variable, but with 10 to 12-day-old cultures there were significantly more transformed plants per replicate (~ 25 embryos) using WPBS medium compared with MS medium (Table 3a, Suppl. Fig. 1k) (P ≤ 0.01). There was no overall significant difference in transformation efficiency with different callus induction periods on WPBS medium (Suppl. Fig. 4), but calli cultured for more than 5 weeks produced virtually no transformants, unless derived from OMM-derived meristems (Table 3c). These cultures produced significantly more transformants per explant and per replicate treatment than immature-embryo-derived calli of a similar age (both P ≤ 0.01), but not per g callus (P ≤ 0.26).

Transformed calli regenerated readily on the RM medium (Fig. 7c) and GUS expression from pBRACT204 was high in transformed plants (Suppl. Figs. 5c, 6d). As a species *L. temulentum* was prone to produce non-regenerating calli and albino shoots, indicating somaclonal variation and a rapid loss of totipotency (Dalton and Thomas 1992). It was preferable therefore to use the youngest cultures possible. However, *L. temulentum* donor plants are susceptible to an endemic bacterial infection which contaminates some immature embryos and is visible after 6 days growth. Donor plants were grown from ‘clean’ embryos, but potential contamination precluded the use of fresh embryos, which were otherwise a relatively transformable explant (Suppl. Fig. 4).

Immersing plated calli with *Agrobacterium* suspension during the infection period (Table 3e) was less effective than vacuum infiltration of *Agrobacterium* suspension into gathered calli, but immersion was a simpler and gentler method.

#### *Brachypodium distachyon*

Immature embryos cultured for up to 7 days were transformable at a low efficiency but were too delicate to handle easily (data not shown). With normal callus induction, the number of transformed plants per replicate (1–2 g callus) of 3-2-1-week and 3-1-week calli grown on WPBS medium was significantly higher than on MS medium (P ≤ 0.01) (Table 3a). The younger, high quality embryogenic callus from the 3-1-week subculture regime also had a higher transformation efficiency than callus from the 4-1-week subculture regime (Table 3d). Transformation using the 3-week-10-day regime was unsuccessful (Suppl. Table 6a).

Transformed plants regenerated readily on the RM medium (Fig. 7d) and GUS was strongly expressed in plants transformed with pBRACT204 (Suppl. Figs. 5d, 6e). Transformation efficiency was significantly higher after vacuum infiltration than after heat shock treatment (P ≤ 0.06) (Table 3f).

#### *Avena sativa, Oryza sativa, Zea mays*

Transformation in *Avena* was problematic as there were many non-transformed escaped plants, whether using paromomycin, hygromycin or PPT/Bialophos for selection (Suppl. Fig. 6h). GUS expressing calli and plants transformed with the construct pTOK233*,* were only recovered when paromomycin rather than hygromycin was used (Suppl. Fig. 5e) and hygromycin selection with pBRACT204 was unsuccessful. In transformations with the *bar* gene, which confers herbicide resistance, *Avena* leaves were assayed with 5 mgl^−1^ PPT and the pH indicator chlorophenol red (Kramer et al. 1993). PPT-resistant leaves turned the medium yellow and the plants were subsequently shown to be PCR positive. PPT resistant plants were recovered after bombardment with the plasmid construct pUBA (*ubi-bar*) (Toki et al. 1992) (Suppl. Fig. 5f lower fifteen leaves), but not using pTF102 (*CaMV35S-bar*), (Suppl. Fig. 5f upper ten leaves), indicating that a strong selectable gene promoter and strong selection was required for successful transformation of this species.

The plasmid pTF102 was also used with *O. sativa*, but GUS expressing plants were only obtained using embryogenic mature-embryo-derived callus because GUS expressing shoot-tip-derived calli were unable to regenerate (Suppl. Fig. 5g). Transformation of *Z. mays* shoot-tip-derived callus with the same plasmid was also unsuccessful as the calli were unable to regenerate, but fresh immature embryos had been previously transformed using the MS based infection and co-cultivation medium (Suppl. Fig. 6g).

#### Other grasses

Three genotypes of *P. arundinacea* were transformed at the first attempt with pBRACT204 using hygromycin selection (Fig. 7e, Suppl. Fig. 6f) and pTOK233 using paromomycin selection (Fig. 7f), although GUS positive plants transformed with pBRACT204 expressed GUS more strongly (Suppl. Fig. 3h), than with pTOK233. *D. cespitosa* was also transformed with pBRACT204 at the first attempt and expressed GUS (Fig. 7g, Suppl. Figs. 5i, 6g).

The plasmid construct pBRACT204 was also successfully used with calli of *F. arundinacea*, *F. rubra* and *A. stolonifera* (Fig. 7h, Suppl. Fig. 5j), induced and grown over three subculture periods. A regime of 5-2-1-weeks or 4-2-2-1-weeks seemed ideal for most species and at least 2 g and up to 30 g of embryogenic callus per ten responding explants could be generated.

In a transformation experiment with *F. arundinacea, L. perenne* and *L. multiflorum* callus, (Fig. 3b) *L. multiflorum* was unsuccessful because the treated calli became overgrown with *Agrobacterium* (Suppl. Table 6g). Another experiment with *L. multiflorum* and *P. pratensis* calli failed due to contamination. Hygromycin, paromomycin or PPT resistant plants were therefore obtained from *Agrobacterium*-mediated transformations of thirteen species using WPBS media (Table 3, Suppl. Table 6) and GUS was expressed in all species except *M. sacchariflorus*. Transformed plants were also obtained by biolistic transformation of *A. sativa*, *F. arundinacea, M. sinensis* and *M. floridulus* (Suppl. Table 6g) using WPBS media. In addition, transgenic hygromycin resistant *L. temulentum, F. arundinacea* and *B. distachyon* cultures were re-bombarded with additional GOI using the *nptII* marker gene and paromomycin selection, as in Buanafina et al. 2015 (Suppl. Table 6g).

### WBPS medium increases transformed plant regeneration and rooting

The RM medium (Table 2) was an effective plant regeneration medium but was improved further when based on WPBS rather than MS media. Calli of sixteen species regenerated well and calli of the thirteen transformed species developed shoots and roots under selection before transfer to WPBS-A based MSO or liquid MS1.5P (*Miscanthus*) maintenance/rooting medium (Suppl. Fig. 6 a–g). Rooted transformed plants of all the species were successfully transferred to soil (Suppl. Fig. 7 a–f).

## Discussion

### Formulation of WPBS medium

The compounds added to MS medium to produce WPBS medium, were either already present in MS medium at lower concentrations, or have been used previously as single additives, to improve embryogenic growth in specific species as described in the introduction.

While the composition of WPBS medium may appear complex, in effect it is easy to make as the additives are simply combined in a stock solution and added to MS medium with 75% macro-elements before autoclaving or filter-sterilisation. The only complication is that myo-inositol needs to be omitted from callus induction medium as it can reduce *Agrobacterium* infectivity (Zhang et al. 2013), while Nawapan et al. (2009) found 2 mM copper sulphate reduced *Agrobacterium* growth. Hence both myo-inositol and copper sulphate are omitted from WPBS based infection and co-cultivation media.

### Callus induction, growth and subculture regimes

WPBS media were successfully used across sixteen species to produce suitable embryogenic calli for transformation, with only minor variations in the auxin and cytokinin concentrations in callus induction and selection media and with additional proline for *Miscanthus* species.

The use of WPBS medium reduced the time that tissues were in culture, which is particularly important in minimising somaclonal variation. This was achieved by decreasing the number of sub-cultures required to produce callus, particularly for *Brachypodium* transformation, while in *L.temulentum* it would be possible and probably preferable to transform fresh immature embryos if sterility could be ensured.

In *Avena*, the wide range of embryo maturity in panicles means that few embryos per panicle are suitable for culture. Shoot tips from sterile seedlings have been used Gasparis and Nadolska-Orczyk (2015), but de-husking seeds for sterilisation is time-consuming. Maqbool et al. (2002) developed OMM medium to induce meristematic clusters and were able to successfully directly transform them. While this was not repeatable in our laboratory, we found that proliferated meristems, particularly in *Avena*, but also in *Festuca* and *Lolium*, greatly increased the amount of embryogenic callus produced per explant and this approach may well be transferable to other species. However, in *Oryza* and *Z. mays*, regenerative embryogenic calli were produced only from embryos.

Using WPBS medium, transformable calli derived from *Oryza* embryos or shoot tips of perennial grass species could be grown and used within 9 to 12 weeks, although embryogenic callus induction in *Miscanthus* species remained slow compared with other genera. However, once established, *Miscanthus* calli grew well and remained regenerative for at least 8 months.

Quantitative determination of callus growth led to the conclusion that sub-culturing more than 0.5 g of callus to a 90 mm Petri-dish containing 25 ml medium reduced callus growth rates. This probably represents the maximum plating density for callus of most species. Transformation efficiency was also greatly reduced by using calli older than 7 days from the last subculture, even in relatively slow growing species such as *Miscanthus* and *Avena*. In conclusion any callus induction regime should aim to produce calli within 3 months, sub-culture no more than 0.5 g callus per Petri-dish and transform callus no older than 7 days.

### Transformation protocol

Using WPBS medium, plant transformation was improved in every species tested, either by increasing callus growth or decreasing the time in culture although significance was sometimes difficult to demonstrate with the transformation stage. However, overall the increased percentage of plants produced per gram of callus using WPBS based media across six species was significantly higher.

Unsuccessful transformations of *L. multiflorum* and *P. pratensis* were due to contamination and of *Z. mays* due to using shoot-tip-derived callus, but thirteen other species were transformed using the same basic protocol and WPBS-based media to induce calli, transform, select and regenerate plants. *P. arundinacea* and *D. cespitosa* calli were transformed in this way at the first attempt. The only differences were in callus induction explant, plant growth regulators for callus growth and sub-culture regime. The same infection, co-cultivation and regeneration media were highly effective in every species, although transformability and regenerative ability naturally differed between species and genotypes.

It is hoped that the use of this WPBS-based media and this general protocol may help to improve the general efficiency of grass and cereal transformation.

## Electronic supplementary material

Below is the link to the electronic supplementary material.

Supplementary file1 (PPTX 28235 kb)

Supplementary file2 (PPTX 89 kb)

Supplementary file3 (DOCX 14 kb)

## Abbreviations

BAP: 6-Benzylaminopurine
cv: Cultivar
2,4-D: 2,4-Dichlorophenoxyacetic acid
FW: Fresh weight
GOI: Genes of interest
MS: Murashige and Skoog
MS1.5P: *Miscanthus* maintenance/rooting medium
MSO: General maintenance/rooting medium
NAA: Naphthalene acetic acid
NS: Not significant
OMM: *Avena* meristem medium
PIG: Particle inflow gun
PPT: Phosphinothricin
RM: Regeneration medium
WPBS: Welsh Plant Breeding Station
