# Supplementary material for: A reformulation of Murashige and Skoog medium (WPBS medium) improves embryogenesis, morphogenesis and transformation efficiency in temperate and tropical grasses and cereals
Source: Plant Cell Tissue Organ Cult. 2020 Feb 19;141(2):257–73. doi: 10.1007/s11240-020-01784-8 (PMC7145791; doi:10.1007/s11240-020-01784-8)
Supplement: Supplementary file 1 — Supplementary file1 (PPTX 28235 kb) [file 11240_2020_1784_MOESM1_ESM.pptx]

## Slide 1
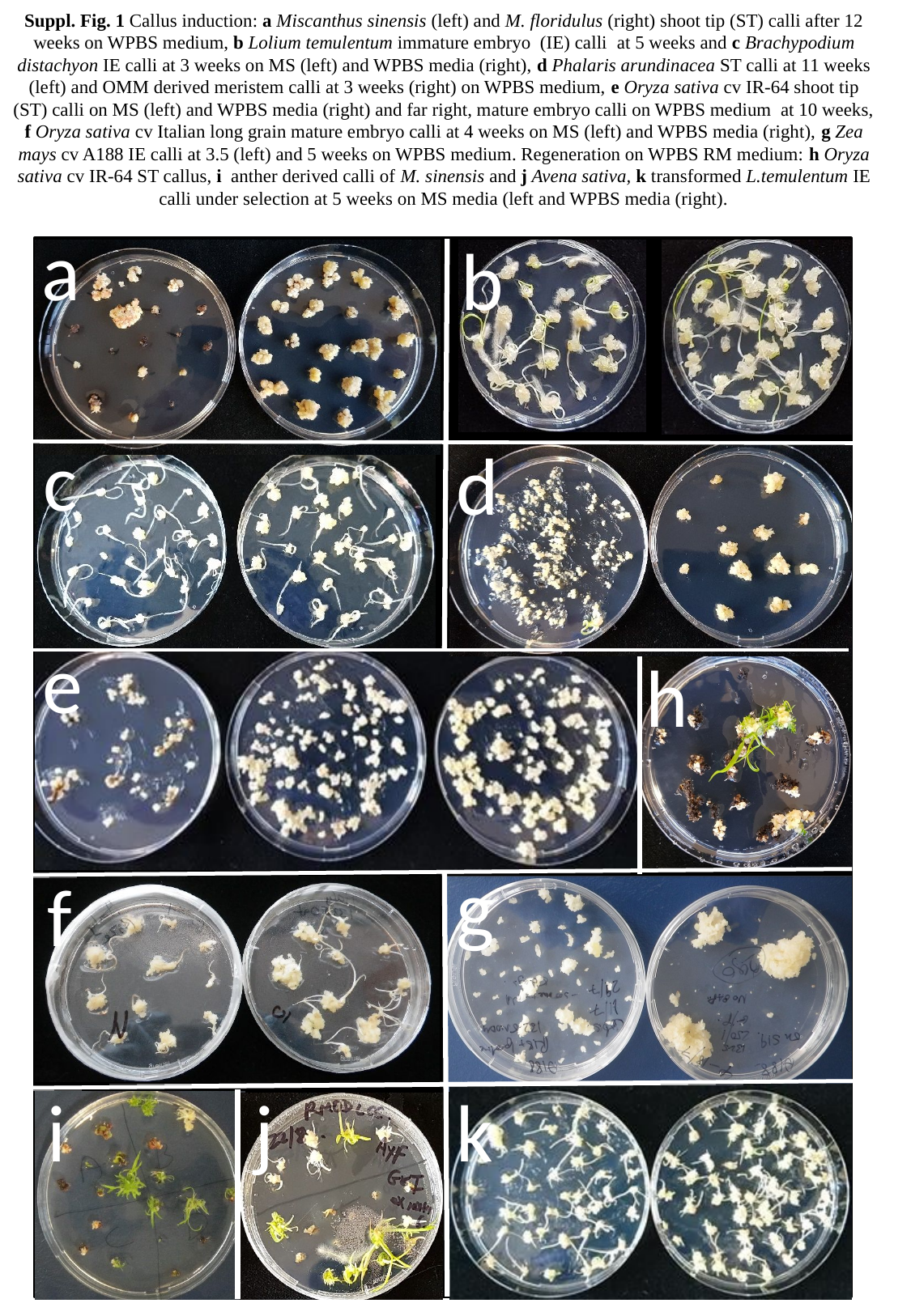

Suppl. Fig. 1 Callus induction: a Miscanthus sinensis (left) and M. floridulus (right) shoot tip (ST) calli after 12 weeks on WPBS medium, b Lolium temulentum immature embryo (IE) calli at 5 weeks and c Brachypodium distachyon IE calli at 3 weeks on MS (left) and WPBS media (right), d Phalaris arundinacea ST calli at 11 weeks (left) and OMM derived meristem calli at 3 weeks (right) on WPBS medium, e Oryza sativa cv IR-64 shoot tip (ST) calli on MS (left) and WPBS media (right) and far right, mature embryo calli on WPBS medium at 10 weeks, f Oryza sativa cv Italian long grain mature embryo calli at 4 weeks on MS (left) and WPBS media (right), g Zea mays cv A188 IE calli at 3.5 (left) and 5 weeks on WPBS medium. Regeneration on WPBS RM medium: h Oryza sativa cv IR-64 ST callus, i anther derived calli of M. sinensis and j Avena sativa, k transformed L.temulentum IE calli under selection at 5 weeks on MS media (left and WPBS media (right).
a
b
c
d
e
h
g
f
i
j
k

## Slide 2
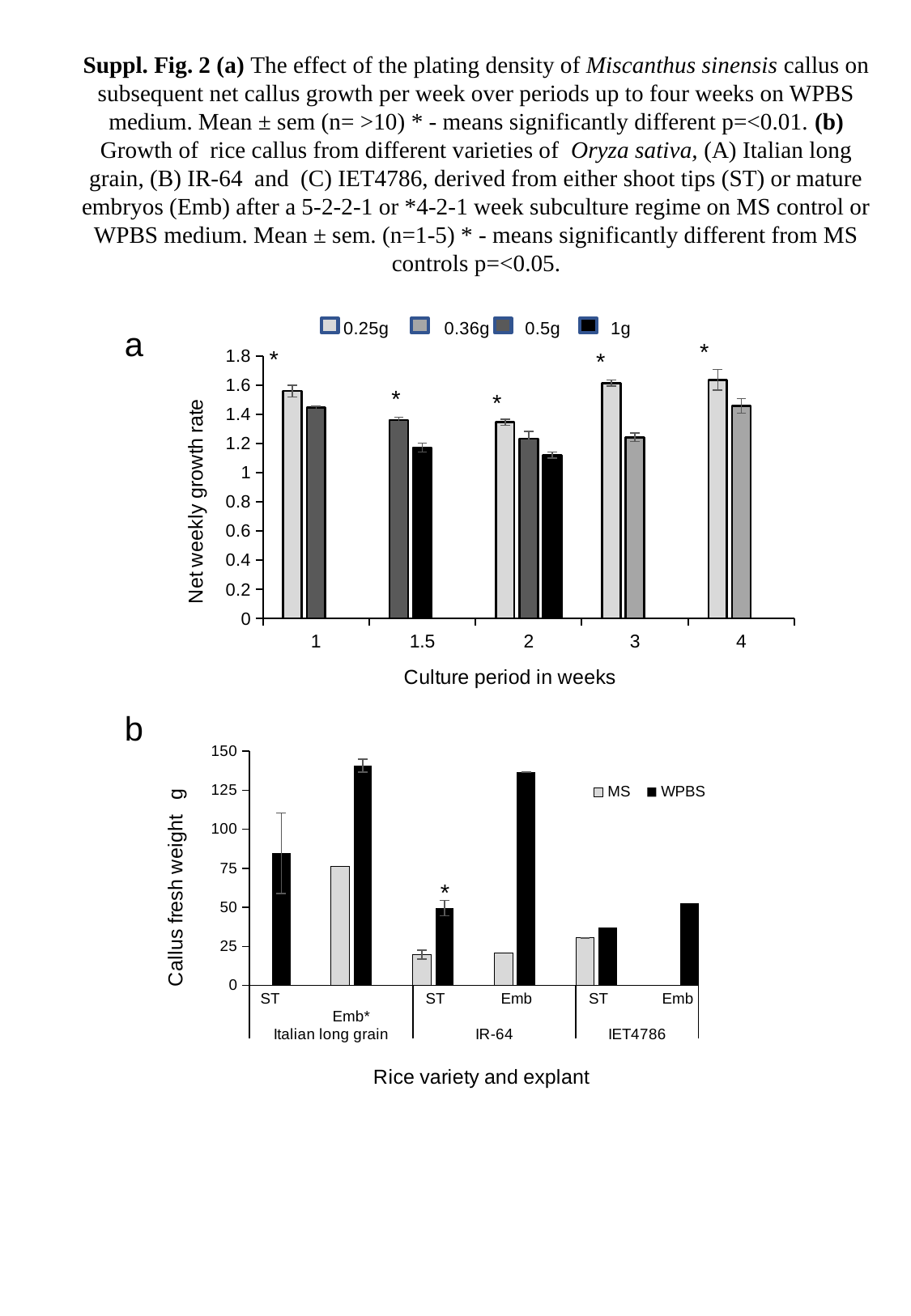

Suppl. Fig. 2 (a) The effect of the plating density of Miscanthus sinensis callus on subsequent net callus growth per week over periods up to four weeks on WPBS medium. Mean ± sem (n= >10) * - means significantly different p=<0.01. (b) Growth of rice callus from different varieties of Oryza sativa, (A) Italian long grain, (B) IR-64 and (C) IET4786, derived from either shoot tips (ST) or mature embryos (Emb) after a 5-2-2-1 or *4-2-1 week subculture regime on MS control or WPBS medium. Mean ± sem. (n=1-5) * - means significantly different from MS controls p=<0.05.
### Chart: 0.25g 0.36g 0.5g 1g
| Category | 0.25g | 0.5g | 1g |
|---|---|---|---|
| 1 | 1.56 | 1.4466666666666668 | None |
| 1.5 | 1.3613397945220116 | 1.1720134339314823 | None |
| 2 | 1.3459999999999999 | 1.233070178112395 | 1.1205092149461615 |
| 3 | 1.6146101692708152 | 1.2432613908491101 | None |
| 4 | 1.6373333333333335 | 1.4576612903225805 | None |a
b
b
### Chart
| Category | MS | WPBS |
|---|---|---|
| ST | None | 84.8 |
| | None | None |
| Emb* | 76.14 | 140.7 |
| | None | None |
| ST | 19.75 | 49.54 |
| | None | None |
| Emb | 21.0 | 136.75 |
| | None | None |
| ST | 30.42 | 37.29 |
| | None | None |
| Emb | None | 52.5 |

## Slide 3
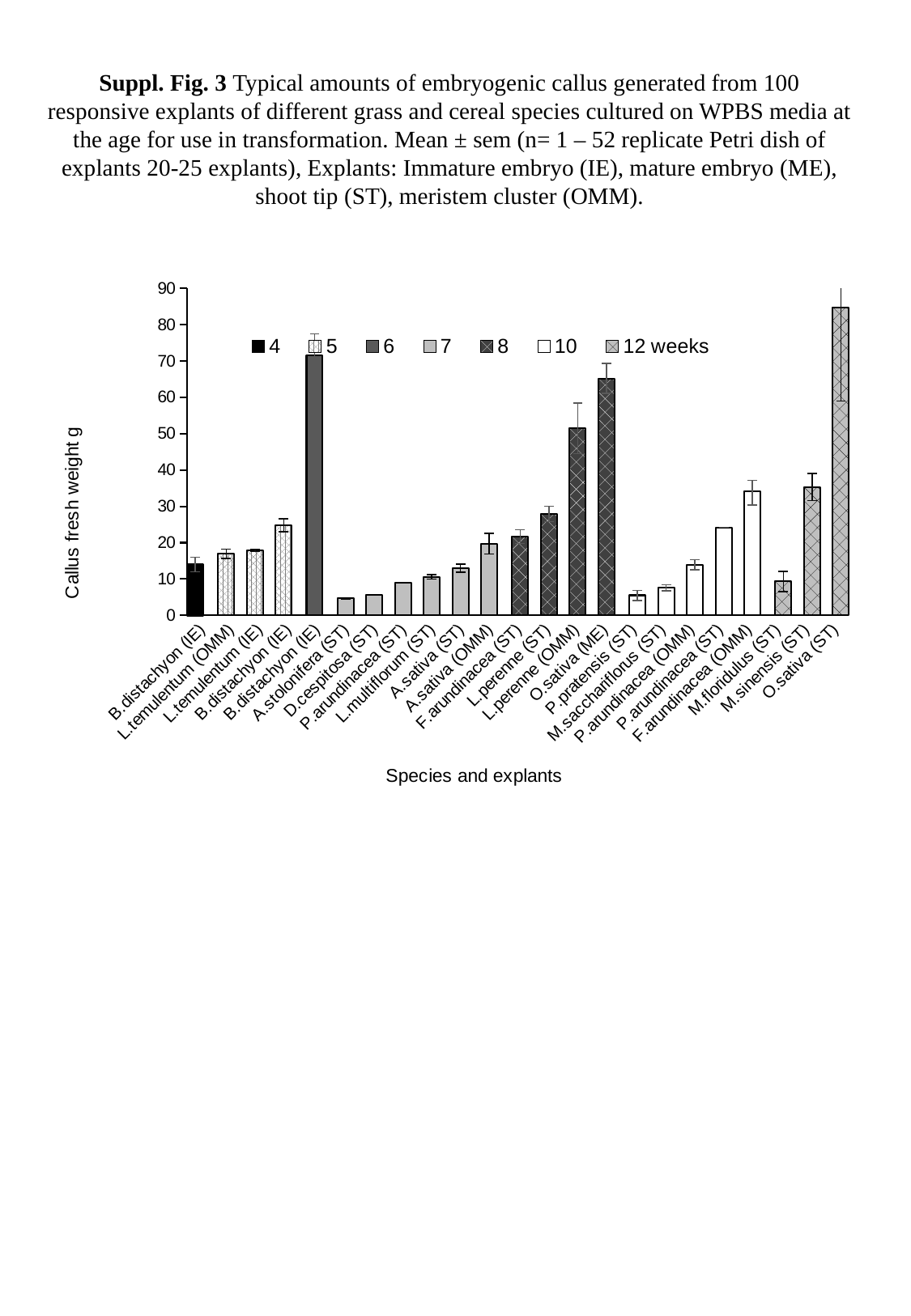

Suppl. Fig. 3 Typical amounts of embryogenic callus generated from 100 responsive explants of different grass and cereal species cultured on WPBS media at the age for use in transformation. Mean ± sem (n= 1 – 52 replicate Petri dish of explants 20-25 explants), Explants: Immature embryo (IE), mature embryo (ME), shoot tip (ST), meristem cluster (OMM).
### Chart
| Category | 4 | 5 | 6 | 7 | 8 | 10 | 12 weeks |
|---|---|---|---|---|---|---|---|
| B.distachyon (IE) | 14.014462754798888 | None | None | None | None | None | None |
| L.temulentum (OMM) | None | 16.934346282372598 | None | None | None | None | None |
| L.temulentum (IE) | None | 17.9 | None | None | None | None | None |
| B.distachyon (IE) | None | 24.83060287864388 | None | None | None | None | None |
| B.distachyon (IE) | None | None | 71.6 | None | None | None | None |
| A.stolonifera (ST) | None | None | None | 4.65 | None | None | None |
| D.cespitosa (ST) | None | None | None | 5.7 | None | None | None |
| P.arundinacea (ST) | None | None | None | 9.0 | None | None | None |
| L.multiflorum (ST) | None | None | None | 10.6 | None | None | None |
| A.sativa (ST) | None | None | None | 13.0 | None | None | None |
| A.sativa (OMM) | None | None | None | 19.74 | None | None | None |
| F.arundinacea (ST) | None | None | None | None | 21.684492753623186 | None | None |
| L.perenne (ST) | None | None | None | None | 27.896428571428572 | None | None |
| L.perenne (OMM) | None | None | None | None | 51.46666666666666 | None | None |
| O.sativa (ME) | None | None | None | None | 65.2 | None | None |
| P.pratensis (ST) | None | None | None | None | None | 5.5 | None |
| M.sacchariflorus (ST) | None | None | None | None | None | 7.6 | None |
| P.arundinacea (OMM) | None | None | None | None | None | 13.9166666666667 | None |
| P.arundinacea (ST) | None | None | None | None | None | 24.2 | None |
| F.arundinacea (OMM) | None | None | None | None | None | 34.16321789321789 | None |
| M.floridulus (ST) | None | None | None | None | None | None | 9.3 |
| M.sinensis (ST) | None | None | None | None | None | None | 35.3 |
| O.sativa (ST) | None | None | None | None | None | None | 84.80357142857143 |

## Slide 4
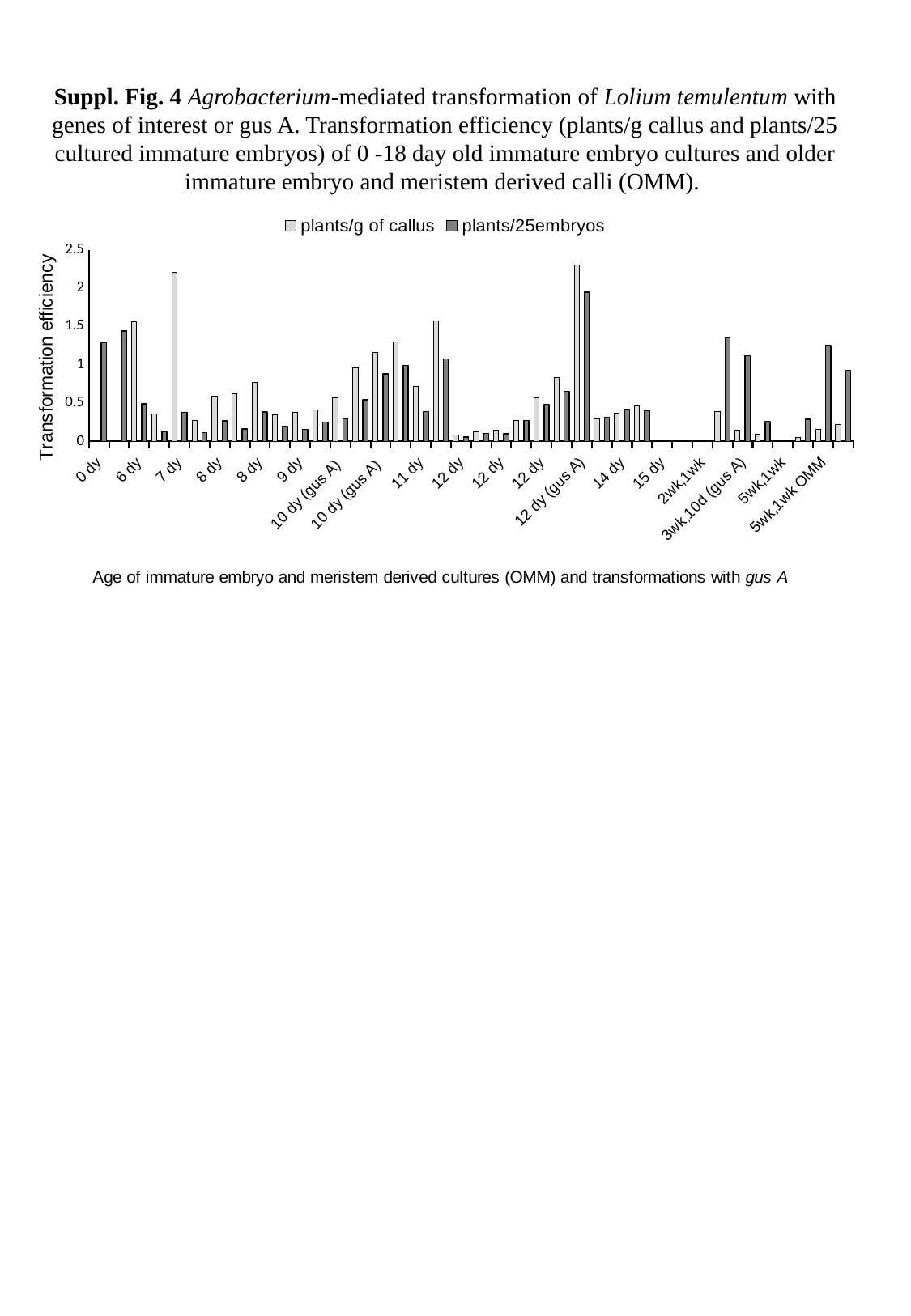

Suppl. Fig. 4 Agrobacterium-mediated transformation of Lolium temulentum with genes of interest or gus A. Transformation efficiency (plants/g callus and plants/25 cultured immature embryos) of 0 -18 day old immature embryo cultures and older immature embryo and meristem derived calli (OMM).
### Chart
| Category | plants/g of callus | plants/25embryos |
|---|---|---|
| 0 dy | 0.0 | 1.287375415282392 |
| 0 dy | 0.0 | 1.4385692068429239 |
| 6 dy | 1.5625 | 0.4901960784313725 |
| 7 dy | 0.3623188405797102 | 0.12987012987012989 |
| 7 dy | 2.2099447513812156 | 0.3773584905660377 |
| 8 dy | 0.2717391304347826 | 0.11709601873536299 |
| 8 dy | 0.5889281507656066 | 0.26709401709401714 |
| 8 dy | 0.6172839506172839 | 0.16233766233766236 |
| 8 dy | 0.7650273224043715 | 0.3863134657836645 |
| 9 dy | 0.35252643948296125 | 0.1928020565552699 |
| 9 dy | 0.38167938931297707 | 0.15723270440251572 |
| 10 dy | 0.40567951318458423 | 0.25316455696202533 |
| 10 dy (gus A) | 0.5681818181818182 | 0.30120481927710846 |
| 10 dy (gus A) | 0.9615384615384615 | 0.5434782608695652 |
| 10 dy (gus A) | 1.1565468814539446 | 0.8838383838383838 |
| 10 dy (gus A) | 1.3011152416356877 | 0.9943181818181818 |
| 11 dy | 0.7173601147776184 | 0.3894080996884735 |
| 11 dy | 1.5736766809728182 | 1.0784313725490198 |
| 12 dy | 0.08779631255487269 | 0.05938242280285035 |
| 12 dy | 0.1251564455569462 | 0.10245901639344264 |
| 12 dy | 0.14992503748125938 | 0.09842519685039369 |
| 12 dy | 0.27578599007170435 | 0.27533039647577096 |
| 12 dy | 0.5649717514124294 | 0.4807692307692307 |
| 12 dy (gus A) | 0.8356545961002786 | 0.6521739130434783 |
| 12 dy (gus A) | 2.298850574712644 | 1.948051948051948 |
| 13 dy | 0.29350104821802936 | 0.30755711775043937 |
| 14 dy | 0.3690036900369003 | 0.41436464088397795 |
| 14 dy | 0.4685408299866131 | 0.4004576659038902 |
| 15 dy | 0.0 | 0.0 |
| 18 dy | 0.0 | 0.0 |
| 2wk,1wk | 0.0 | 0.0 |
| 3wk,1wk OMM | 0.3849855630413859 | 1.3513513513513513 |
| 3wk,10d (gus A) | 0.1454192922927775 | 1.1194029850746268 |
| 4wk,1wk | 0.09053870529651425 | 0.2590673575129534 |
| 5wk,1wk | 0.0 | 0.0 |
| 5wk,1wk OMM | 0.048520135856380396 | 0.29069767441860467 |
| 5wk,1wk OMM | 0.15366884364195157 | 1.25 |
| 5wk,1wk OMM | 0.22189349112426035 | 0.9259259259259258 |

## Slide 5
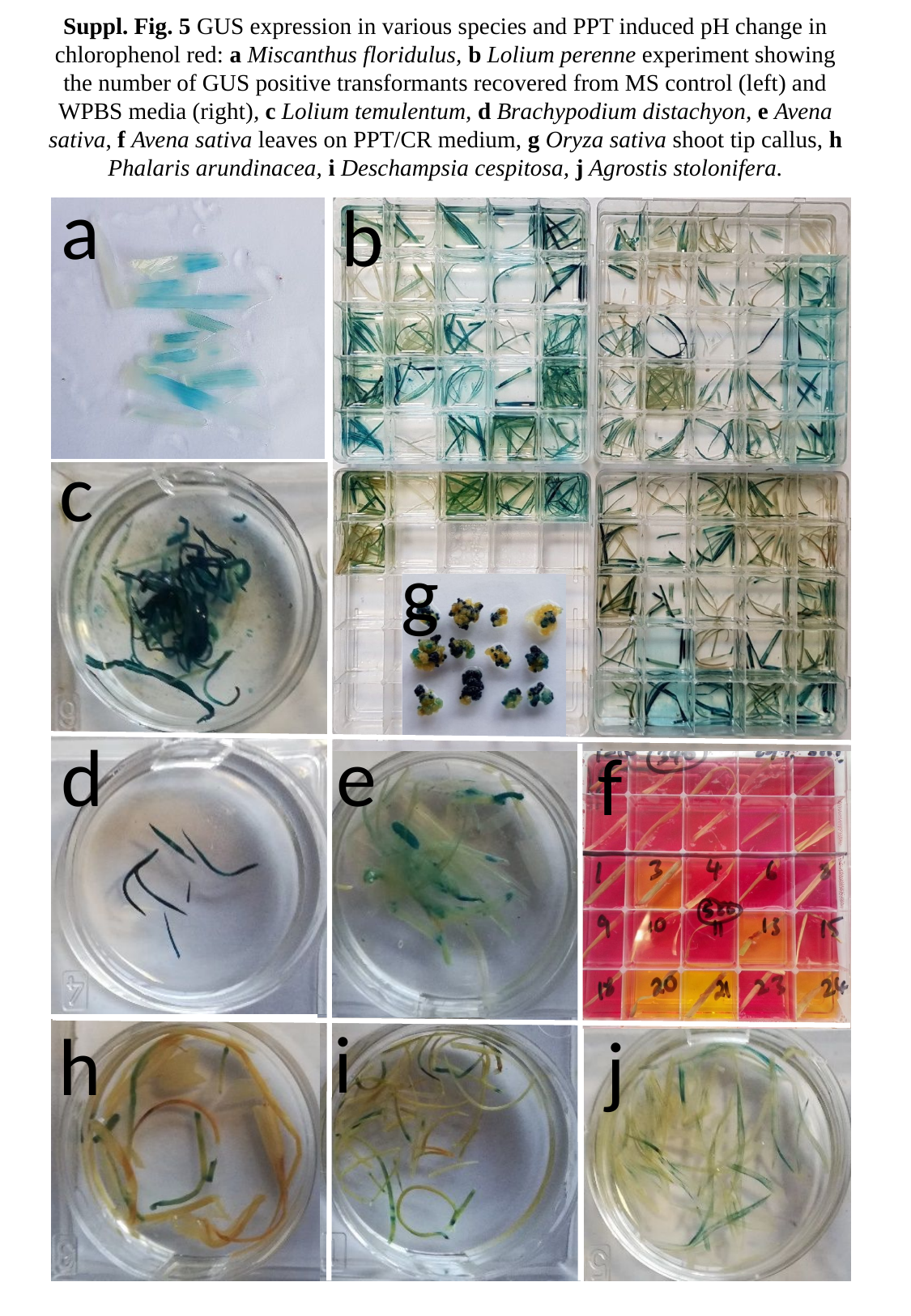

Suppl. Fig. 5 GUS expression in various species and PPT induced pH change in chlorophenol red: a Miscanthus floridulus, b Lolium perenne experiment showing the number of GUS positive transformants recovered from MS control (left) and WPBS media (right), c Lolium temulentum, d Brachypodium distachyon, e Avena sativa, f Avena sativa leaves on PPT/CR medium, g Oryza sativa shoot tip callus, h Phalaris arundinacea, i Deschampsia cespitosa, j Agrostis stolonifera.
a
b
c
d
e
f
i
h
j
g

## Slide 6
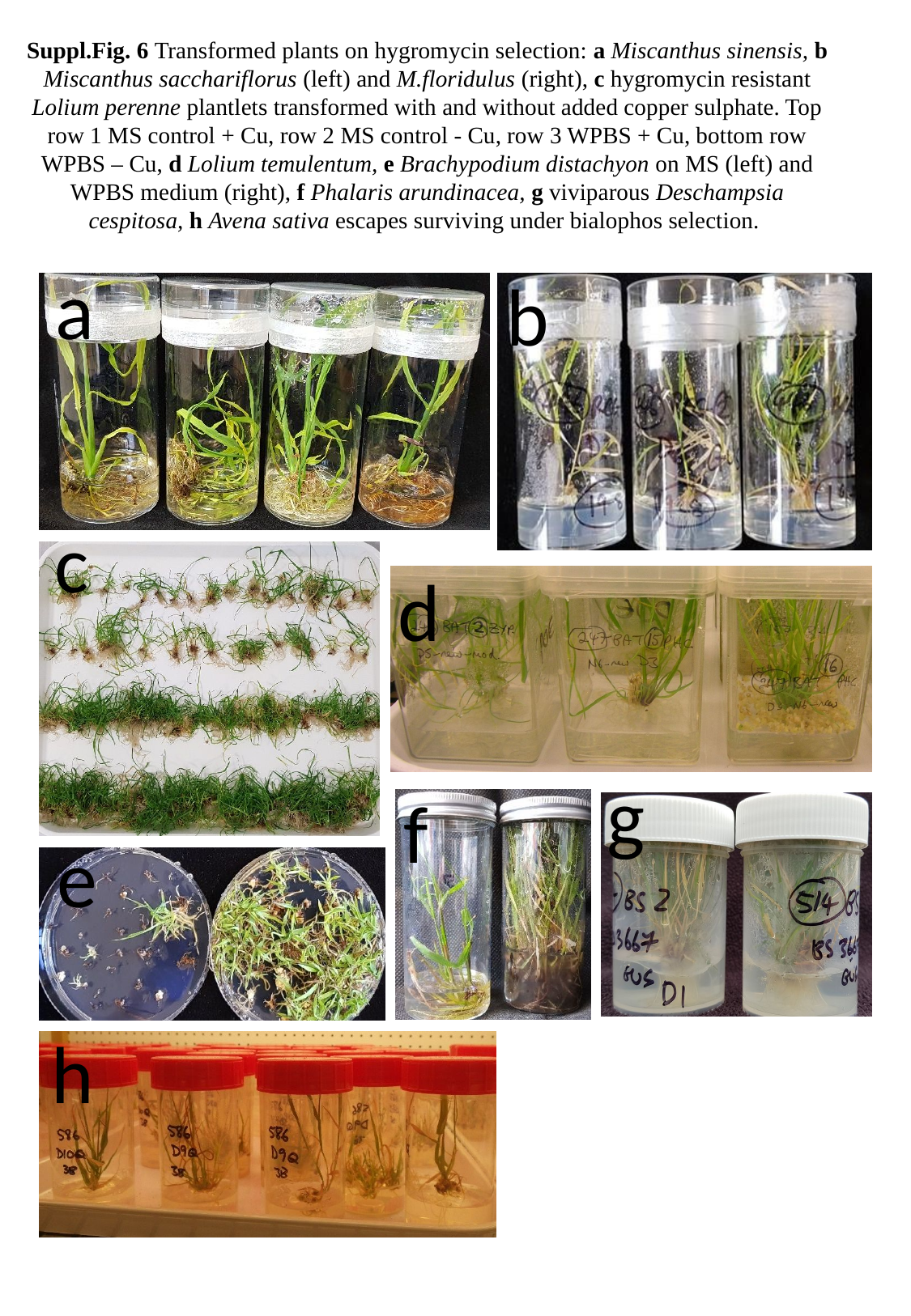

Suppl.Fig. 6 Transformed plants on hygromycin selection: a Miscanthus sinensis, b Miscanthus sacchariflorus (left) and M.floridulus (right), c hygromycin resistant Lolium perenne plantlets transformed with and without added copper sulphate. Top row 1 MS control + Cu, row 2 MS control - Cu, row 3 WPBS + Cu, bottom row WPBS – Cu, d Lolium temulentum, e Brachypodium distachyon on MS (left) and WPBS medium (right), f Phalaris arundinacea, g viviparous Deschampsia cespitosa, h Avena sativa escapes surviving under bialophos selection.
a
b
c
d
g
f
e
h

## Slide 7
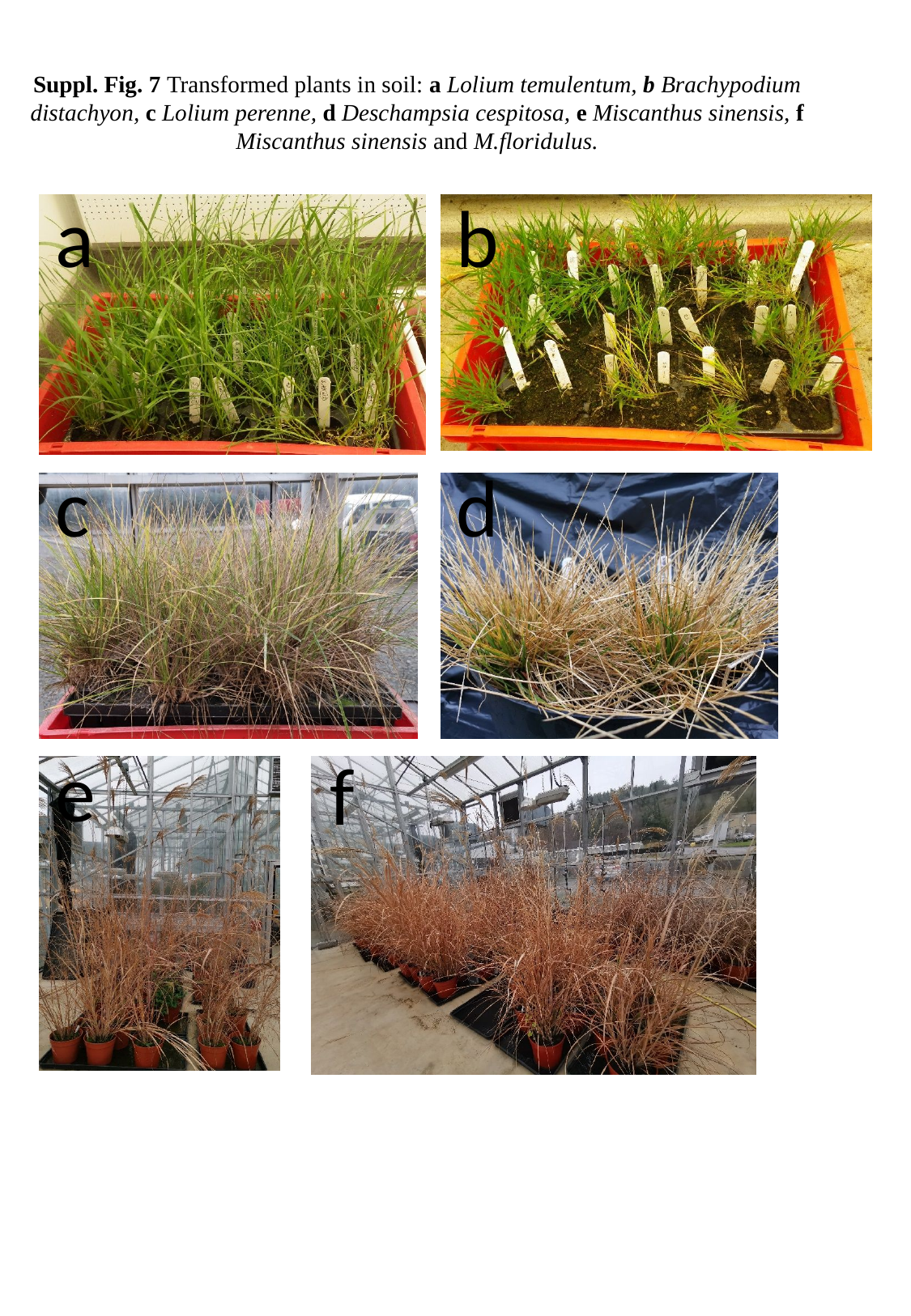

Suppl. Fig. 7 Transformed plants in soil: a Lolium temulentum, b Brachypodium distachyon, c Lolium perenne, d Deschampsia cespitosa, e Miscanthus sinensis, f Miscanthus sinensis and M.floridulus.
a
b
c
d
e
f
