# Supplementary material for: A reformulation of Murashige and Skoog medium (WPBS medium) improves embryogenesis, morphogenesis and transformation efficiency in temperate and tropical grasses and cereals
Source: Plant Cell Tissue Organ Cult. 2020 Feb 19;141(2):257–73. doi: 10.1007/s11240-020-01784-8 (PMC7145791; doi:10.1007/s11240-020-01784-8)
Supplement: Supplementary file 2 — Supplementary file2 (PPTX 89 kb) [file 11240_2020_1784_MOESM2_ESM.pptx]

## Slide 1
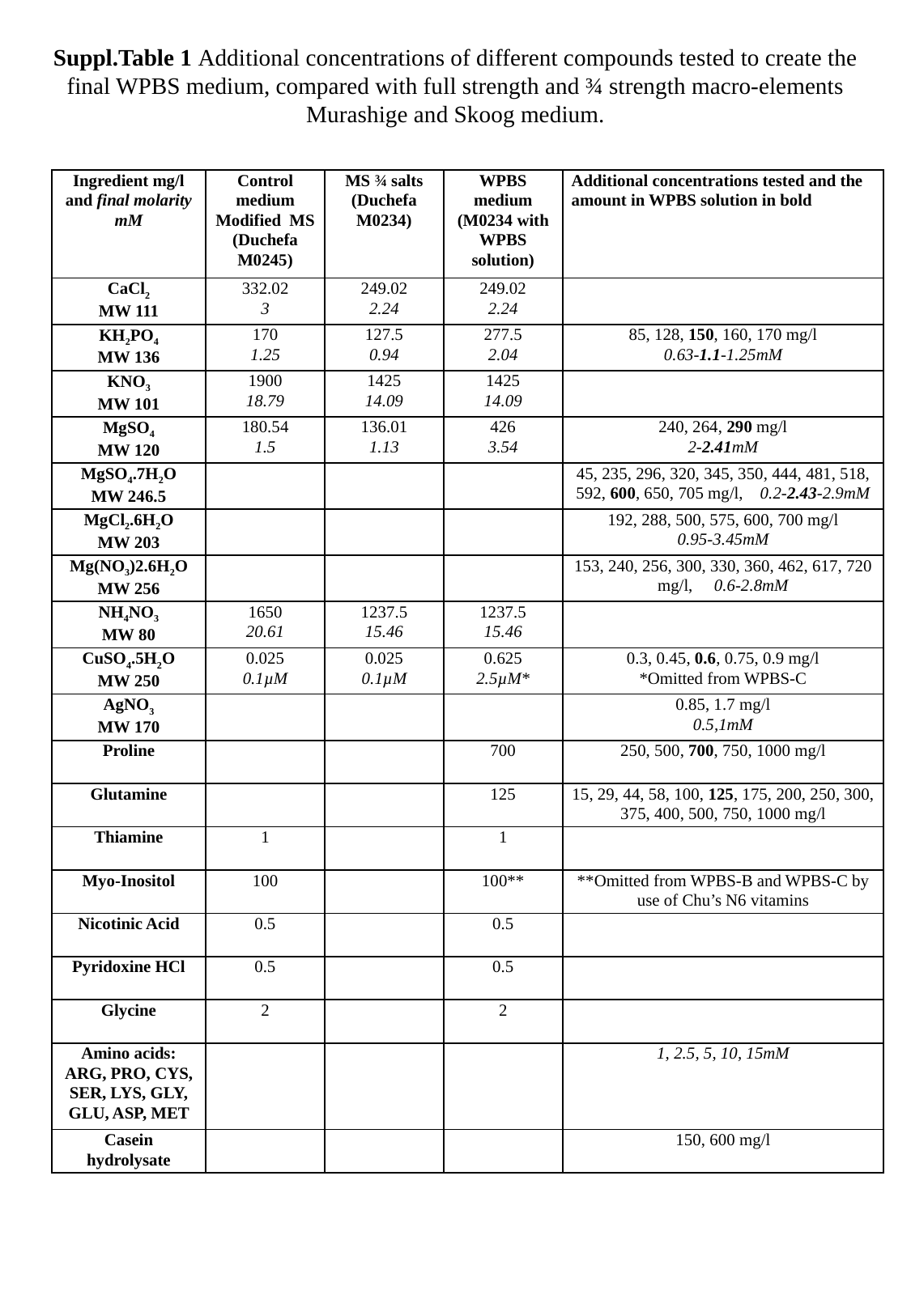

Suppl.Table 1 Additional concentrations of different compounds tested to create the final WPBS medium, compared with full strength and ¾ strength macro-elements Murashige and Skoog medium.
| Ingredient mg/l and final molarity mM | Control medium Modified MS (Duchefa M0245) | MS ¾ salts (Duchefa M0234) | WPBS medium (M0234 with WPBS solution) | Additional concentrations tested and the amount in WPBS solution in bold |
| --- | --- | --- | --- | --- |
| CaCl2 MW 111 | 332.02 3 | 249.02 2.24 | 249.02 2.24 | |
| KH2PO4 MW 136 | 170 1.25 | 127.5 0.94 | 277.5 2.04 | 85, 128, 150, 160, 170 mg/l 0.63-1.1-1.25mM |
| KNO3 MW 101 | 1900 18.79 | 1425 14.09 | 1425 14.09 | |
| MgSO4 MW 120 | 180.54 1.5 | 136.01 1.13 | 426 3.54 | 240, 264, 290 mg/l 2-2.41mM |
| MgSO4.7H2O MW 246.5 | | | | 45, 235, 296, 320, 345, 350, 444, 481, 518, 592, 600, 650, 705 mg/l, 0.2-2.43-2.9mM |
| MgCl2.6H2O MW 203 | | | | 192, 288, 500, 575, 600, 700 mg/l 0.95-3.45mM |
| Mg(NO3)2.6H2O MW 256 | | | | 153, 240, 256, 300, 330, 360, 462, 617, 720 mg/l, 0.6-2.8mM |
| NH4NO3 MW 80 | 1650 20.61 | 1237.5 15.46 | 1237.5 15.46 | |
| CuSO4.5H2O MW 250 | 0.025 0.1µM | 0.025 0.1µM | 0.625 2.5µM\* | 0.3, 0.45, 0.6, 0.75, 0.9 mg/l \*Omitted from WPBS-C |
| AgNO3 MW 170 | | | | 0.85, 1.7 mg/l 0.5,1mM |
| Proline | | | 700 | 250, 500, 700, 750, 1000 mg/l |
| Glutamine | | | 125 | 15, 29, 44, 58, 100, 125, 175, 200, 250, 300, 375, 400, 500, 750, 1000 mg/l |
| Thiamine | 1 | | 1 | |
| Myo-Inositol | 100 | | 100\*\* | \*\*Omitted from WPBS-B and WPBS-C by use of Chu’s N6 vitamins |
| Nicotinic Acid | 0.5 | | 0.5 | |
| Pyridoxine HCl | 0.5 | | 0.5 | |
| Glycine | 2 | | 2 | |
| Amino acids: ARG, PRO, CYS, SER, LYS, GLY, GLU, ASP, MET | | | | 1, 2.5, 5, 10, 15mM |
| Casein hydrolysate | | | | 150, 600 mg/l |

## Slide 2
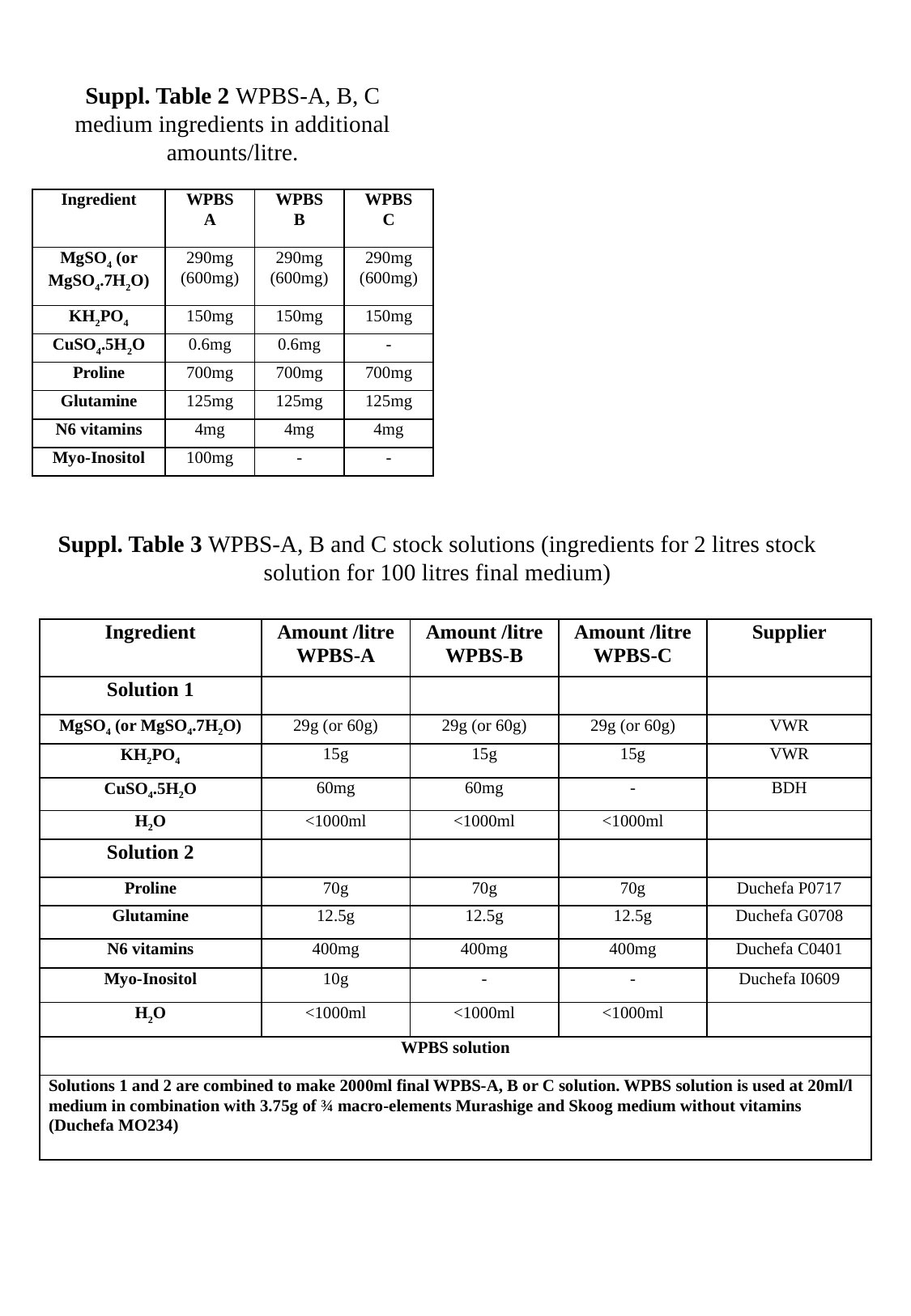

Suppl. Table 2 WPBS-A, B, C medium ingredients in additional amounts/litre.
| Ingredient | WPBS A | WPBS B | WPBS C |
| --- | --- | --- | --- |
| MgSO4 (or MgSO4.7H2O) | 290mg (600mg) | 290mg (600mg) | 290mg (600mg) |
| KH2PO4 | 150mg | 150mg | 150mg |
| CuSO4.5H2O | 0.6mg | 0.6mg | - |
| Proline | 700mg | 700mg | 700mg |
| Glutamine | 125mg | 125mg | 125mg |
| N6 vitamins | 4mg | 4mg | 4mg |
| Myo-Inositol | 100mg | - | - |
Suppl. Table 3 WPBS-A, B and C stock solutions (ingredients for 2 litres stock solution for 100 litres final medium)
| Ingredient | Amount /litre WPBS-A | Amount /litre WPBS-B | Amount /litre WPBS-C | Supplier |
| --- | --- | --- | --- | --- |
| Solution 1 | | | | |
| MgSO4 (or MgSO4.7H2O) | 29g (or 60g) | 29g (or 60g) | 29g (or 60g) | VWR |
| KH2PO4 | 15g | 15g | 15g | VWR |
| CuSO4.5H2O | 60mg | 60mg | - | BDH |
| H2O | <1000ml | <1000ml | <1000ml | |
| Solution 2 | | | | |
| Proline | 70g | 70g | 70g | Duchefa P0717 |
| Glutamine | 12.5g | 12.5g | 12.5g | Duchefa G0708 |
| N6 vitamins | 400mg | 400mg | 400mg | Duchefa C0401 |
| Myo-Inositol | 10g | - | - | Duchefa I0609 |
| H2O | <1000ml | <1000ml | <1000ml | |
| WPBS solution | | | | |
| Solutions 1 and 2 are combined to make 2000ml final WPBS-A, B or C solution. WPBS solution is used at 20ml/l medium in combination with 3.75g of ¾ macro-elements Murashige and Skoog medium without vitamins (Duchefa MO234) | | | | |

## Slide 3
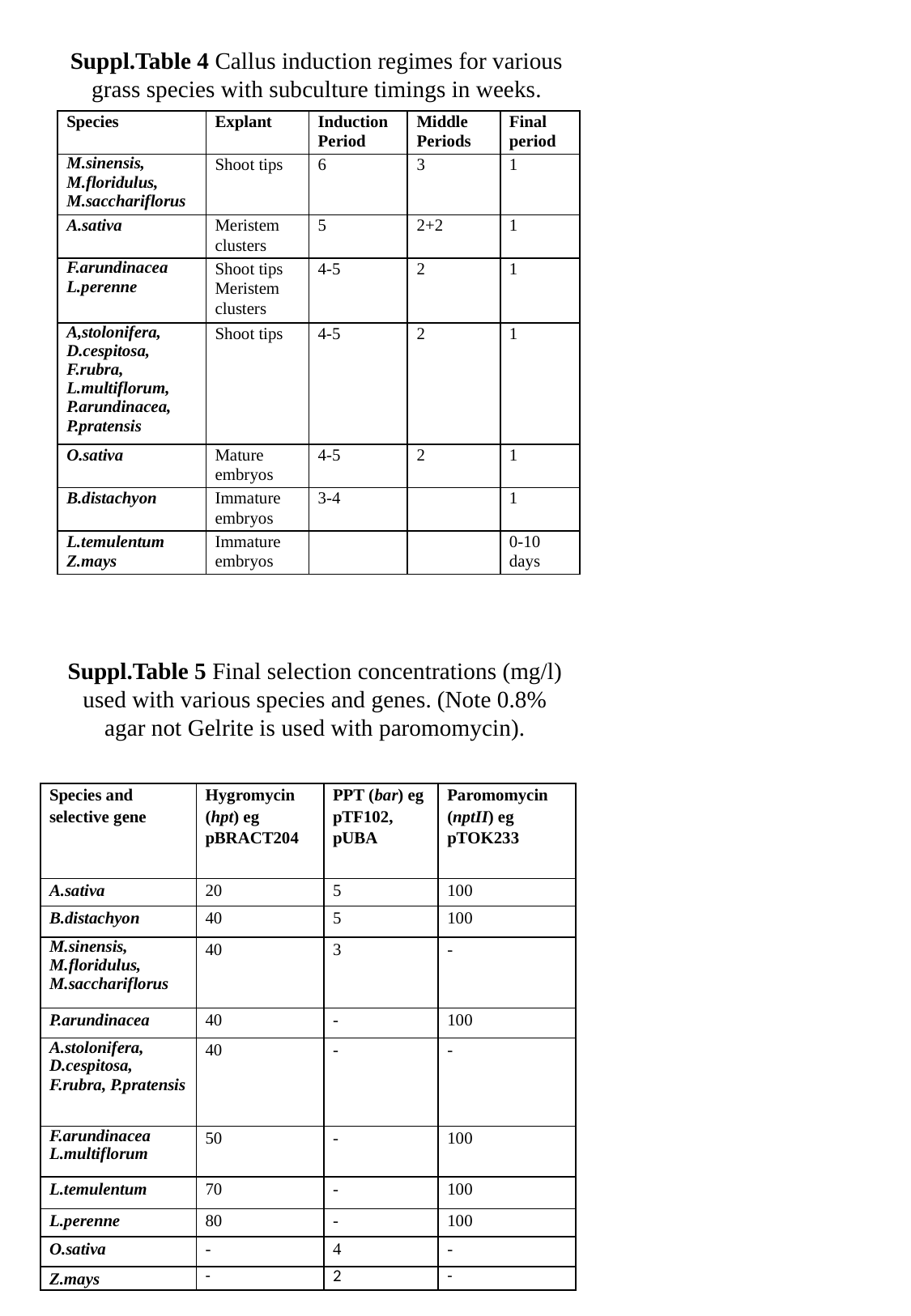

Suppl.Table 4 Callus induction regimes for various grass species with subculture timings in weeks.
| Species | Explant | Induction Period | Middle Periods | Final period |
| --- | --- | --- | --- | --- |
| M.sinensis, M.floridulus, M.sacchariflorus | Shoot tips | 6 | 3 | 1 |
| A.sativa | Meristem clusters | 5 | 2+2 | 1 |
| F.arundinacea L.perenne | Shoot tips Meristem clusters | 4-5 | 2 | 1 |
| A,stolonifera, D.cespitosa, F.rubra, L.multiflorum, P.arundinacea, P.pratensis | Shoot tips | 4-5 | 2 | 1 |
| O.sativa | Mature embryos | 4-5 | 2 | 1 |
| B.distachyon | Immature embryos | 3-4 | | 1 |
| L.temulentum Z.mays | Immature embryos | | | 0-10 days |
Suppl.Table 5 Final selection concentrations (mg/l) used with various species and genes. (Note 0.8% agar not Gelrite is used with paromomycin).
| Species and selective gene | Hygromycin (hpt) eg pBRACT204 | PPT (bar) eg pTF102, pUBA | Paromomycin (nptII) eg pTOK233 |
| --- | --- | --- | --- |
| A.sativa | 20 | 5 | 100 |
| B.distachyon | 40 | 5 | 100 |
| M.sinensis, M.floridulus, M.sacchariflorus | 40 | 3 | - |
| P.arundinacea | 40 | - | 100 |
| A.stolonifera, D.cespitosa, F.rubra, P.pratensis | 40 | - | - |
| F.arundinacea L.multiflorum | 50 | - | 100 |
| L.temulentum | 70 | - | 100 |
| L.perenne | 80 | - | 100 |
| O.sativa | - | 4 | - |
| Z.mays | - | 2 | - |

## Slide 4
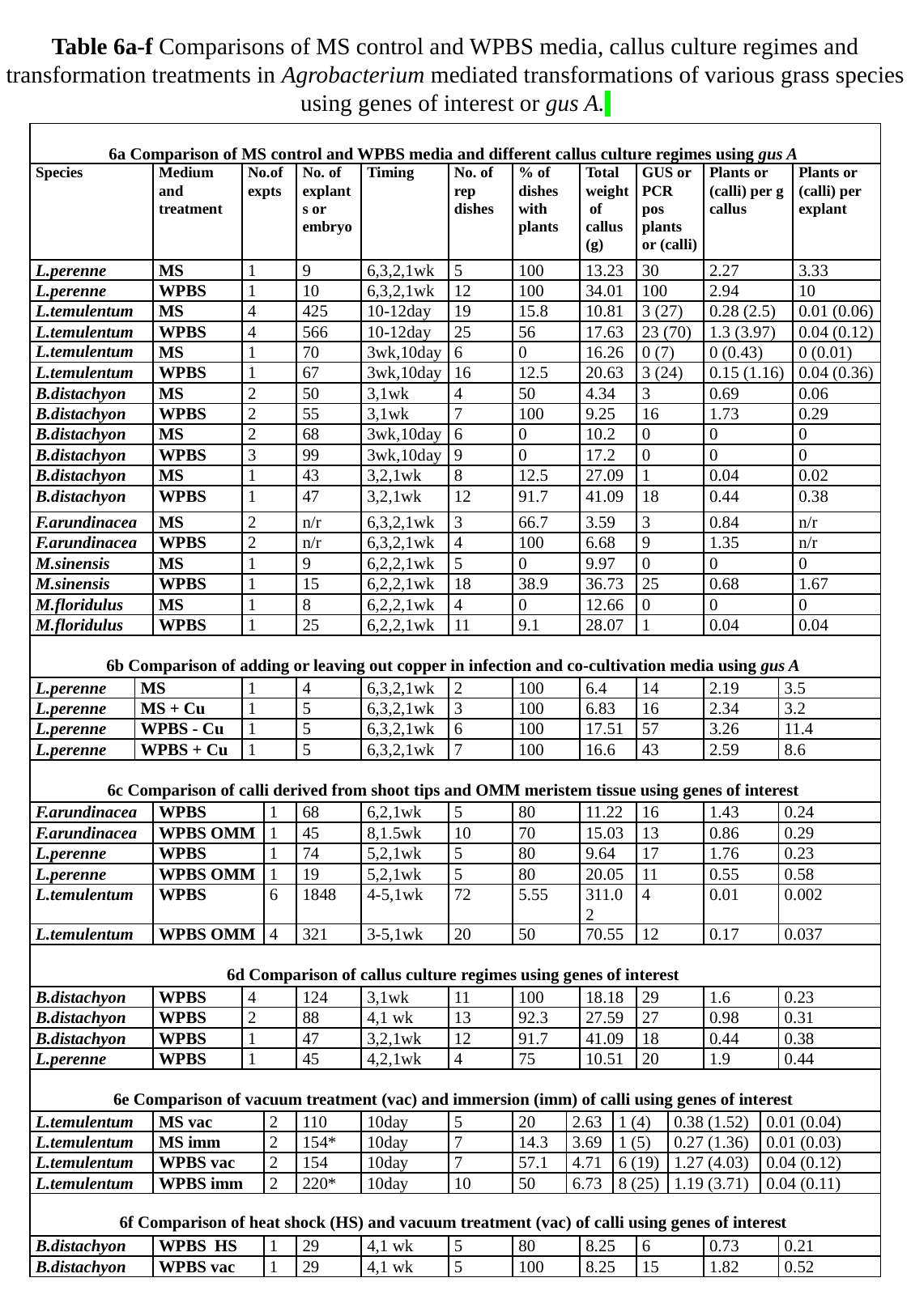

Table 6a-f Comparisons of MS control and WPBS media, callus culture regimes and transformation treatments in Agrobacterium mediated transformations of various grass species using genes of interest or gus A.
| 6a Comparison of MS control and WPBS media and different callus culture regimes using gus A | | | | | | | | | | | | | | | | | |
| --- | --- | --- | --- | --- | --- | --- | --- | --- | --- | --- | --- | --- | --- | --- | --- | --- | --- |
| Species | Medium and treatment | Medium and treatment | No.of expts | No.of expts | No. of explants or embryo | Timing | No. of rep dishes | % of dishes with plants | | Total weight of callus (g) | | GUS or PCR pos plants or (calli) | | Plants or (calli) per g callus | | Plants or (calli) per explant | Plants or (calli) per explant |
| L.perenne | MS | MS | 1 | 1 | 9 | 6,3,2,1wk | 5 | 100 | | 13.23 | | 30 | | 2.27 | | 3.33 | 3.33 |
| L.perenne | WPBS | WPBS | 1 | 1 | 10 | 6,3,2,1wk | 12 | 100 | | 34.01 | | 100 | | 2.94 | | 10 | 10 |
| L.temulentum | MS | MS | 4 | 4 | 425 | 10-12day | 19 | 15.8 | | 10.81 | | 3 (27) | | 0.28 (2.5) | | 0.01 (0.06) | 0.01 (0.06) |
| L.temulentum | WPBS | WPBS | 4 | 4 | 566 | 10-12day | 25 | 56 | | 17.63 | | 23 (70) | | 1.3 (3.97) | | 0.04 (0.12) | 0.04 (0.12) |
| L.temulentum | MS | MS | 1 | 1 | 70 | 3wk,10day | 6 | 0 | | 16.26 | | 0 (7) | | 0 (0.43) | | 0 (0.01) | 0 (0.01) |
| L.temulentum | WPBS | WPBS | 1 | 1 | 67 | 3wk,10day | 16 | 12.5 | | 20.63 | | 3 (24) | | 0.15 (1.16) | | 0.04 (0.36) | 0.04 (0.36) |
| B.distachyon | MS | MS | 2 | 2 | 50 | 3,1wk | 4 | 50 | | 4.34 | | 3 | | 0.69 | | 0.06 | 0.06 |
| B.distachyon | WPBS | WPBS | 2 | 2 | 55 | 3,1wk | 7 | 100 | | 9.25 | | 16 | | 1.73 | | 0.29 | 0.29 |
| B.distachyon | | MS | 2 | 2 | 68 | 3wk,10day | 6 | 0 | | 10.2 | | 0 | | 0 | | 0 | 0 |
| B.distachyon | | WPBS | 3 | 3 | 99 | 3wk,10day | 9 | 0 | | 17.2 | | 0 | | 0 | | 0 | 0 |
| B.distachyon | MS | MS | 1 | 1 | 43 | 3,2,1wk | 8 | 12.5 | | 27.09 | | 1 | | 0.04 | | 0.02 | 0.02 |
| B.distachyon | WPBS | WPBS | 1 | 1 | 47 | 3,2,1wk | 12 | 91.7 | | 41.09 | | 18 | | 0.44 | | 0.38 | 0.38 |
| F.arundinacea | MS | MS | 2 | 2 | n/r | 6,3,2,1wk | 3 | 66.7 | | 3.59 | | 3 | | 0.84 | | n/r | n/r |
| F.arundinacea | WPBS | WPBS | 2 | 2 | n/r | 6,3,2,1wk | 4 | 100 | | 6.68 | | 9 | | 1.35 | | n/r | n/r |
| M.sinensis | MS | MS | 1 | 1 | 9 | 6,2,2,1wk | 5 | 0 | | 9.97 | | 0 | | 0 | | 0 | 0 |
| M.sinensis | WPBS | WPBS | 1 | 1 | 15 | 6,2,2,1wk | 18 | 38.9 | | 36.73 | | 25 | | 0.68 | | 1.67 | 1.67 |
| M.floridulus | MS | MS | 1 | 1 | 8 | 6,2,2,1wk | 4 | 0 | | 12.66 | | 0 | | 0 | | 0 | 0 |
| M.floridulus | WPBS | WPBS | 1 | 1 | 25 | 6,2,2,1wk | 11 | 9.1 | | 28.07 | | 1 | | 0.04 | | 0.04 | 0.04 |
| 6b Comparison of adding or leaving out copper in infection and co-cultivation media using gus A | | | | | | | | | | | | | | | | | |
| L.perenne | MS | | 1 | | 4 | 6,3,2,1wk | 2 | 100 | | 6.4 | | 14 | | 2.19 | | 3.5 | |
| L.perenne | MS + Cu | | 1 | | 5 | 6,3,2,1wk | 3 | 100 | | 6.83 | | 16 | | 2.34 | | 3.2 | |
| L.perenne | WPBS - Cu | | 1 | | 5 | 6,3,2,1wk | 6 | 100 | | 17.51 | | 57 | | 3.26 | | 11.4 | |
| L.perenne | WPBS + Cu | | 1 | | 5 | 6,3,2,1wk | 7 | 100 | | 16.6 | | 43 | | 2.59 | | 8.6 | |
| 6c Comparison of calli derived from shoot tips and OMM meristem tissue using genes of interest | | | | | | | | | | | | | | | | | |
| F.arundinacea | WPBS | WPBS | 1 | 1 | 68 | 6,2,1wk | 5 | 80 | | 11.22 | | 16 | | 1.43 | | 0.24 | |
| F.arundinacea | WPBS OMM | WPBS OMM | 1 | 1 | 45 | 8,1.5wk | 10 | 70 | | 15.03 | | 13 | | 0.86 | | 0.29 | |
| L.perenne | WPBS | WPBS | 1 | 1 | 74 | 5,2,1wk | 5 | 80 | | 9.64 | | 17 | | 1.76 | | 0.23 | |
| L.perenne | WPBS OMM | WPBS OMM | 1 | 1 | 19 | 5,2,1wk | 5 | 80 | | 20.05 | | 11 | | 0.55 | | 0.58 | |
| L.temulentum | WPBS | WPBS | 6 | 6 | 1848 | 4-5,1wk | 72 | 5.55 | | 311.02 | | 4 | | 0.01 | | 0.002 | |
| L.temulentum | WPBS OMM | WPBS OMM | 4 | 4 | 321 | 3-5,1wk | 20 | 50 | | 70.55 | | 12 | | 0.17 | | 0.037 | |
| 6d Comparison of callus culture regimes using genes of interest | | | | | | | | | | | | | | | | | |
| B.distachyon | WPBS | WPBS | 4 | | 124 | 3,1wk | 11 | 100 | | 18.18 | | 29 | | 1.6 | | 0.23 | |
| B.distachyon | WPBS | WPBS | 2 | | 88 | 4,1 wk | 13 | 92.3 | | 27.59 | | 27 | | 0.98 | | 0.31 | |
| B.distachyon | WPBS | WPBS | 1 | | 47 | 3,2,1wk | 12 | 91.7 | | 41.09 | | 18 | | 0.44 | | 0.38 | |
| L.perenne | WPBS | WPBS | 1 | | 45 | 4,2,1wk | 4 | 75 | | 10.51 | | 20 | | 1.9 | | 0.44 | |
| 6e Comparison of vacuum treatment (vac) and immersion (imm) of calli using genes of interest | | | | | | | | | | | | | | | | | |
| L.temulentum | MS vacuum | MS vac | 2 | 2 | 110 | 10day | 5 | 20 | 2.63 | 2.63 | 1 (4) | 1 (4) | 0.38 (1.52) | 0.38 (1.52) | 0.01 (0.04) | 0.01 (0.04) | |
| L.temulentum | MS bathe | MS imm | 2 | 2 | 154\* | 10day | 7 | 14.3 | 3.69 | 3.69 | 1 (5) | 1 (5) | 0.27 (1.36) | 0.27 (1.36) | 0.01 (0.03) | 0.01 (0.03) | |
| L.temulentum | WPBS vacuum | WPBS vac | 2 | 2 | 154 | 10day | 7 | 57.1 | 4.71 | 4.71 | 6 (19) | 6 (19) | 1.27 (4.03) | 1.27 (4.03) | 0.04 (0.12) | 0.04 (0.12) | |
| L.temulentum | WPBS bathe | WPBS imm | 2 | 2 | 220\* | 10day | 10 | 50 | 6.73 | 6.73 | 8 (25) | 8 (25) | 1.19 (3.71) | 1.19 (3.71) | 0.04 (0.11) | 0.04 (0.11) | |
| 6f Comparison of heat shock (HS) and vacuum treatment (vac) of calli using genes of interest | | | | | | | | | | | | | | | | | |
| B.distachyon | WPBS HS | WPBS HS | 1 | 1 | 29 | 4,1 wk | 5 | 80 | | 8.25 | | 6 | | 0.73 | | 0.21 | |
| B.distachyon | WPBS vacuum | WPBS vac | 1 | 1 | 29 | 4,1 wk | 5 | 100 | | 8.25 | | 15 | | 1.82 | | 0.52 | |

## Slide 5
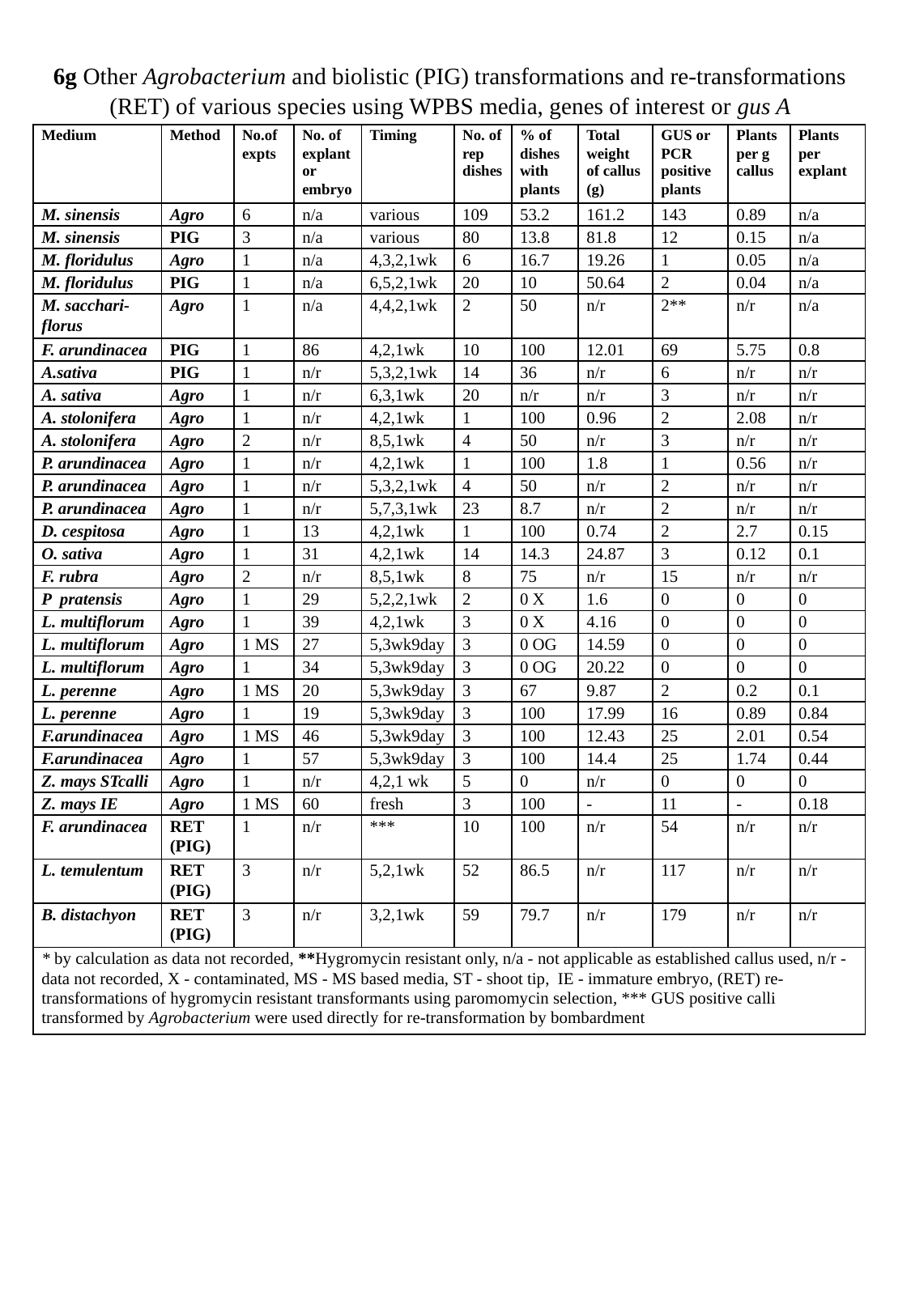

6g Other Agrobacterium and biolistic (PIG) transformations and re-transformations (RET) of various species using WPBS media, genes of interest or gus A
| Medium | Method | No.of expts | No. of explant or embryo | Timing | No. of rep dishes | % of dishes with plants | Total weight of callus (g) | GUS or PCR positive plants | Plants per g callus | Plants per explant |
| --- | --- | --- | --- | --- | --- | --- | --- | --- | --- | --- |
| M. sinensis | Agro | 6 | n/a | various | 109 | 53.2 | 161.2 | 143 | 0.89 | n/a |
| M. sinensis | PIG | 3 | n/a | various | 80 | 13.8 | 81.8 | 12 | 0.15 | n/a |
| M. floridulus | Agro | 1 | n/a | 4,3,2,1wk | 6 | 16.7 | 19.26 | 1 | 0.05 | n/a |
| M. floridulus | PIG | 1 | n/a | 6,5,2,1wk | 20 | 10 | 50.64 | 2 | 0.04 | n/a |
| M. sacchari-florus | Agro | 1 | n/a | 4,4,2,1wk | 2 | 50 | n/r | 2\*\* | n/r | n/a |
| F. arundinacea | PIG | 1 | 86 | 4,2,1wk | 10 | 100 | 12.01 | 69 | 5.75 | 0.8 |
| A.sativa | PIG | 1 | n/r | 5,3,2,1wk | 14 | 36 | n/r | 6 | n/r | n/r |
| A. sativa | Agro | 1 | n/r | 6,3,1wk | 20 | n/r | n/r | 3 | n/r | n/r |
| A. stolonifera | Agro | 1 | n/r | 4,2,1wk | 1 | 100 | 0.96 | 2 | 2.08 | n/r |
| A. stolonifera | Agro | 2 | n/r | 8,5,1wk | 4 | 50 | n/r | 3 | n/r | n/r |
| P. arundinacea | Agro | 1 | n/r | 4,2,1wk | 1 | 100 | 1.8 | 1 | 0.56 | n/r |
| P. arundinacea | Agro | 1 | n/r | 5,3,2,1wk | 4 | 50 | n/r | 2 | n/r | n/r |
| P. arundinacea | Agro | 1 | n/r | 5,7,3,1wk | 23 | 8.7 | n/r | 2 | n/r | n/r |
| D. cespitosa | Agro | 1 | 13 | 4,2,1wk | 1 | 100 | 0.74 | 2 | 2.7 | 0.15 |
| O. sativa | Agro | 1 | 31 | 4,2,1wk | 14 | 14.3 | 24.87 | 3 | 0.12 | 0.1 |
| F. rubra | Agro | 2 | n/r | 8,5,1wk | 8 | 75 | n/r | 15 | n/r | n/r |
| P pratensis | Agro | 1 | 29 | 5,2,2,1wk | 2 | 0 X | 1.6 | 0 | 0 | 0 |
| L. multiflorum | Agro | 1 | 39 | 4,2,1wk | 3 | 0 X | 4.16 | 0 | 0 | 0 |
| L. multiflorum | Agro | 1 MS | 27 | 5,3wk9day | 3 | 0 OG | 14.59 | 0 | 0 | 0 |
| L. multiflorum | Agro | 1 | 34 | 5,3wk9day | 3 | 0 OG | 20.22 | 0 | 0 | 0 |
| L. perenne | Agro | 1 MS | 20 | 5,3wk9day | 3 | 67 | 9.87 | 2 | 0.2 | 0.1 |
| L. perenne | Agro | 1 | 19 | 5,3wk9day | 3 | 100 | 17.99 | 16 | 0.89 | 0.84 |
| F.arundinacea | Agro | 1 MS | 46 | 5,3wk9day | 3 | 100 | 12.43 | 25 | 2.01 | 0.54 |
| F.arundinacea | Agro | 1 | 57 | 5,3wk9day | 3 | 100 | 14.4 | 25 | 1.74 | 0.44 |
| Z. mays STcalli | Agro | 1 | n/r | 4,2,1 wk | 5 | 0 | n/r | 0 | 0 | 0 |
| Z. mays IE | Agro | 1 MS | 60 | fresh | 3 | 100 | - | 11 | - | 0.18 |
| F. arundinacea | RET (PIG) | 1 | n/r | \*\*\* | 10 | 100 | n/r | 54 | n/r | n/r |
| L. temulentum | RET (PIG) | 3 | n/r | 5,2,1wk | 52 | 86.5 | n/r | 117 | n/r | n/r |
| B. distachyon | RET (PIG) | 3 | n/r | 3,2,1wk | 59 | 79.7 | n/r | 179 | n/r | n/r |
| \* by calculation as data not recorded, \*\*Hygromycin resistant only, n/a - not applicable as established callus used, n/r - data not recorded, X - contaminated, MS - MS based media, ST - shoot tip, IE - immature embryo, (RET) re-transformations of hygromycin resistant transformants using paromomycin selection, \*\*\* GUS positive calli transformed by Agrobacterium were used directly for re-transformation by bombardment | | | | | | | | | | |
