# Supplementary material for: A reformulation of Murashige and Skoog medium (WPBS medium) improves embryogenesis, morphogenesis and transformation efficiency in temperate and tropical grasses and cereals
Source: Plant Cell Tissue Organ Cult. 2020 Feb 19;141(2):257–73. doi: 10.1007/s11240-020-01784-8 (PMC7145791; doi:10.1007/s11240-020-01784-8)
Supplement: Supplementary file 3 — Supplementary file3 (DOCX 14 kb) [file 11240_2020_1784_MOESM3_ESM.docx]

Suppl. Text 1, Rationale for the development of WPBS medium.

The induction of fast-growing, embryogenic callus is a pre-requisite for efficient genetic transformation in grasses and cereals. However, *M.sinensis*, although embryogenic in many genotypes, is normally slow growing despite the beneficial addition of proline, thiamine and low concentrations of 6-benzylaminopurine (BAP) (Dalton 2013). Currently, callus is only transformed with low efficiency (Wang et al 2011, Hwang et al 2014, Yoo et al 2018) and is not yet routine. An ideal grass transformation system such as that devised for *Brachypodium* (Thole and Vain 2012) produces sufficient embryogenic callus tissue for transformation in six weeks. However, most *Miscanthus* explants have barely started to grow in this time scale.

Gramineous cultures have generally been grown on Murashige and Skoog (1962) medium (Wang et al 2001), with maltose being more effective than sucrose as a carbon source for callus induction and growth. (Dalton and Thomas 1992). Further work with *Miscanthus* species has also shown that increased levels of thiamine (Dalton 2013), low concentrations of BAP (Wang et al 2011) and relatively high concentrations of proline are essential in this genus (Holme et al 1997, Dalton 2013, Hwang et al 2014). Low proline concentrations have long been used in callus cultures of maize (Frame et al 2002) and more recently in *Lolium* (Patel et al 2013). Proline and glutamine have been used together in rice (Pawar et al 2015) and in *Miscanthus* callus culture (Yoo et al 2018) and glutamine has been found helpful in *Agrobacterium*-mediated transformation of *L.perenne* (Zhang et al 2013).

Several other compounds have improved embryogenesis in specific species such as copper sulphate with *Poa* (Ha et al 2001), oat (Choi et al 2001) and *Brachypodium* (Thole and Vain 2012), magnesium chloride with *Miscanthus* (Holme et al 1997) and silver nitrate in maize (Frame et al 2002). Pepó and Tóth (2003) demonstrated that phosphate in the form of potassium phosphate was the first mineral to be depleted by *Miscanthus* cultures grown on MS medium. Some culture medium components have also been found to affect plant transformation. Myo-inositol was shown to reduce *Agrobacterium* *tumefaciens* infectivity (Zhang et al 2013) and copper sulphate to reduce *Agrobacterium* growth (Nawapan et al 2009).

Copper sulphate, magnesium chloride and silver nitrate have been shown to improve embryogenic callus growth in specific graminaeous species while potassium phosphate can be rapidly depleted in cultures. The rationale for creating WPBS medium was to compare these four compounds and combine the best to improve *Miscanthus* callus growth.

Silver nitrate was soon discontinued as it had little effect, but the other three compounds independently improved callus growth. Various combinations of the three were compared and the best combination improved callus growth further. The amount of salts in the medium was now quite high however, so the best combination was added to 50%, 75% and 100% strength MS macro-elements to compare growth. The 75% macro-element medium improved growth still further and was used thereafter.

Magnesium chloride was very hygroscopic and difficult to use and chlorine was not generally considered a useful nutrient. Magnesium sulphate was then substituted for magnesium chloride to varying degrees until it was the only magnesium compound. Varying amounts of magnesium nitrate were then substituted for the magnesium sulphate to see the effect of nitrate, but this increased friable growth at the expense of embryogenesis. However, this confirmed that the additional sulphate was helpful.

The medium was originally developed to improve the growth and transformation efficiency of *Miscanthus* callus, but the additives proved beneficial with every other grass tested. Proline was omitted from media used with other grasses. However, oat callus still did not grow very fast and a screen of amino acids showed that proline and glutamine were most helpful. Initially large amounts of glutamine and proline were used, but they were found detrimental to embryogenesis and regeneration. Lower concentrations were more generally helpful. Finally, the copper sulphate concentration was re-examined and increased.

Over one hundred combinations of the various additives were tested and assessed over six years and the final recipe was named WPBS medium to celebrate the 2019 centenary of the Welsh Plant Breeding Station, latterly known as IBERS.
